# Mechanisms of fluid movement into, through and out of the brain: evaluation of the evidence

**DOI:** 10.1186/2045-8118-11-26

**Published:** 2014-12-02

**Authors:** Stephen B Hladky, Margery A Barrand

**Affiliations:** Department of Pharmacology, University of Cambridge, Cambridge, CB2 1PD UK

**Keywords:** Blood-brain barrier, Brain interstitial fluid, Cerebrospinal fluid, Choroid plexus, Convection, Diffusion, Filtration, Periarterial space, Phase contrast magnetic resonance imaging, Secretion

## Abstract

Interstitial fluid (ISF) surrounds the parenchymal cells of the brain and spinal cord while cerebrospinal fluid (CSF) fills the larger spaces within and around the CNS. Regulation of the composition and volume of these fluids is important for effective functioning of brain cells and is achieved by barriers that prevent free exchange between CNS and blood and by mechanisms that secrete fluid of controlled composition into the brain and distribute and reabsorb it. Structures associated with this regular fluid turnover include the choroid plexuses, brain capillaries comprising the blood-brain barrier, arachnoid villi and perineural spaces penetrating the cribriform plate. ISF flow, estimated from rates of removal of markers from the brain, has been thought to reflect rates of fluid secretion across the blood-brain barrier, although this has been questioned because measurements were made under barbiturate anaesthesia possibly affecting secretion and flow and because CSF influx to the parenchyma via perivascular routes may deliver fluid independently of blood-brain barrier secretion. Fluid secretion at the blood-brain barrier is provided by specific transporters that generate solute fluxes so creating osmotic gradients that force water to follow. Any flow due to hydrostatic pressures driving water across the barrier soon ceases unless accompanied by solute transport because water movements modify solute concentrations. CSF is thought to be derived primarily from secretion by the choroid plexuses. Flow rates measured using phase contrast magnetic resonance imaging reveal CSF movements to be more rapid and variable than previously supposed, even implying that under some circumstances *net* flow through the cerebral aqueduct may be reversed with net flow into the third and lateral ventricles. Such reversed flow requires there to be alternative sites for both generation and removal of CSF. Fluorescent tracer analysis has shown that fluid flow can occur from CSF into parenchyma along periarterial spaces. Whether this represents *net* fluid flow and whether there is subsequent flow through the interstitium and *net* flow out of the cortex via perivenous routes, described as glymphatic circulation, remains to be established. Modern techniques have revealed complex fluid movements within the brain. This review provides a critical evaluation of the data.

## Review

1. The context
  1.1 Introduction
  1.2 The anatomy and terminology
  1.3 Functions of the fluids
  1.4 Composition of fluids
  1.5 Anatomical basis of barriers and routes of fluid transfer
2. Basic principles of fluid movements in the brain and lessons from studies on peripheral tissues
  2.1 Diffusion
  2.2 Bulk flow or convection
  2.3 Instances and relative importance of diffusion and bulk flow
  2.4 Transfers by specific transporters
  2.5 Filtration, secretion and absorption
    2.5.1 Filtration
    2.5.1.1 Mechanistic descriptions of filtration
    2.5.2 Secretion and absorption
  2.6 Uses of tracers or marker substances and lessons from the periphery
  2.7 Application of basic principles to blood vessels in the brain
  2.8 Diffusion and convection within the parenchyma
  2.9 Secretion by the choroid plexuses and the blood-brain barrier
3. Formation and removal of CSF and ISF
  3.1 CSF
    3.1.1 Formation of CSF
    3.1.2 Removal of CSF
    3.1.3 Measurement of CSF production rate
  3.2 Measurement of ISF production rate
4. Ongoing approaches to the investigation of brain fluid dynamics
  4.1 Studies of movement of substances and routes of outflow from the brain parenchyma.
    4.1.1 Periarterial spaces as routes of efflux
      4.1.1.1 Evaluation of the proposal that periarterial spaces provide an efflux route for markers
    4.1.2 Extracellular spaces of the arterial smooth muscle layer as routes of efflux
  4.2 Studies of CSF flow and the implications of the flow patterns for sites and rates of production and absorption of CSF and ISF
    4.2.1.1 Measurement of CSF flow by phase contrast magnetic resonance imaging (PC-MRI)
    4.2.1.2 Cyclic variations in CSF flow
    4.2.1.3 Direction of net flow
    4.2.2 Changes in flow in hydrocephalus
      4.2.2.1 Non-communicating hydrocephalus
      4.2.2.2 Communicating hydrocephalus and the possibility of reverse net flow
    4.2.3 CSF flow in infants
    4.2.4 Possible alternative routes for CSF outflow from the ventricles
    4.2.5 Caveats on PC-MRI results for net flow through the aqueduct
  4.3 Recent studies on perivascular routes for entry into and exit from the cortex
    4.3.1 The basis of the glymphatic circulation proposal: evidence from fluorescence imaging studies
    4.3.2 Quantification of influx and efflux using radiotracers
    4.3.3 Influences of aquaporin 4 (AQP4) located on astrocyte endfeet on perivascular flow
    4.3.4 Reassessment of the evidence: alternatives to the glymphatic proposal
  4.4 Studies concerning the influence of sleep and anaesthesia on perivascular fluid flow and interstitial fluid volume
    4.4.1 Changes in flow and volume inferred from rates of delivery of fluorescent markers
    4.4.2 Interstitial fluid volume changes inferred from TMA iontophoresis measurements
    4.4.3 Interstitial fluid volume changes inferred from radiotracer measurements
5. A current view of regulation of the extracellular fluids of the brain and their constant renewal
6. Conclusion

## 1 The context

### 1.1 Introduction

The fluids that surround the brain and bathe the cells within it play several important roles. They provide the most suitable ionic and nutrient microenvironment to allow neurons and associated cells to function correctly: they deliver all the substances the cells require and remove unwanted material and they provide a cushion against physical damage. Hence regulation of composition, volume and turnover of these fluids is vital. To allow this regulation there are barriers that prevent free exchange of material between brain and blood, mechanisms that secrete fluid of controlled composition into the brain, and mechanisms that reabsorb, eventually to blood, the extracellular fluids whatever their composition. Structures associated with this regulation include those generating the fluid, i.e. the choroid plexuses and the brain capillaries that make up the blood-CSF and blood-brain barriers, respectively, and those able to remove the fluid, e.g. the arachnoid villi and the perineural spaces of nerves penetrating the cribriform plate. This review considers the relative contribution of these structures in secreting and removing the fluids and evaluates the evidence for the various ideas of how fluid secreted into the brain may move through the brain parenchyma and thence exit to the periphery.

The traditional view is that the choroid plexuses secrete the major portion of the cerebrospinal fluid (CSF) surrounding the brain while the blood-brain barrier has been thought to make a much smaller contribution to fluid production, generating interstitial fluid (ISF) that drains into CSF. Comparisons between these interfaces with regard to their rates of secretion are considered in sections 3 and 4. Ideas regarding the relative importance of these structures in regulating brain fluid may need to be reassessed and will be discussed further in a later publication. Many of the topics considered in this review have been discussed on previous occasions. The reader interested in accounts of earlier work with references should consult a number of excellent reviews: [1–3] for anatomy and generally [4–14]. Recently, Damkier *et al.*[15] have presented a comprehensive account of the mechanisms of secretion by the choroid plexuses, Pollay [16] has discussed the routes for absorption of CSF and Brinker *et al.*[17] have surveyed topics of current interest about the circulation of CSF. O’Donnell [18] has reviewed the mechanisms at the blood-brain barrier with attention to factors that can regulate or modulate secretion and has considered the changes that occur in focal oedema. Strazielle and Ghersi-Egea [19] have discussed the structure and function of the blood-brain interfaces and Engelhardt and Sorokin [20] and Luissant *et al.*[21] have reviewed the molecular basis and properties of the tight junctions important for barrier function.

### 1.2 The anatomy and terminology

**Figure 1:**
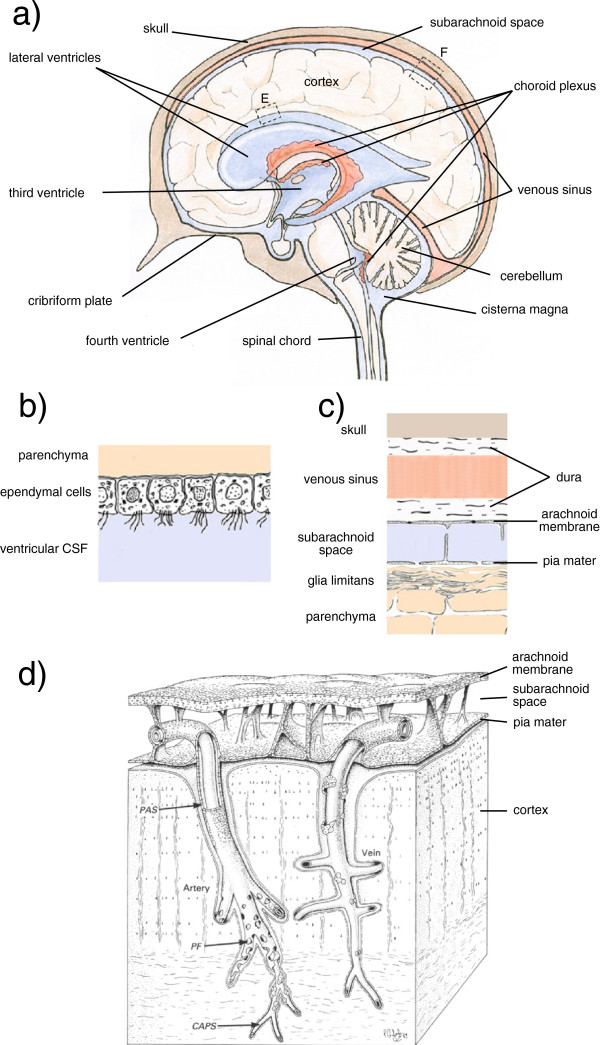
**Structures of the brain considered in this review. a)** Mid-sagittal section from nose to the back of the head incorporating images of the lateral ventricles that lie to each side of the section. CSF filled spaces are shown in blue, blood filled spaces in pale red. The choroid plexuses are shown in darker red. **b)** Enlarged view of surface of a lateral ventricle at (E). **c)** Enlarged view of the cortical surface at (F). The glia limitans is a mat of glial processes. **d)** Drawing showing the relationships between the arachnoid membrane, the pia mater, the subarachnoid space, and the vasculature supplying the cortex. As described by Zhang *et al.* for the artery “the sheath has been cut away to show that the periarterial spaces (PAS) of the intracerebral and extracerebral arteries are in continuity. The layer of pial cells becomes perforated (PF) and incomplete as smooth muscle cells are lost from the smaller branches of the artery. The pial sheath finally disappears as the perivascular spaces are obliterated around capillaries (CAPS). Perivascular spaces around the vein (right of picture) are confluent with the subpial space and only small numbers of pial cells are associated with the vessel wall” [23]. The cortical boundary along these vessels is formed by glial foot-processes. The graphic elements in **a)** and **b)** and in **c)** with minor extensions are taken with permission from Figure one of Strazielle *et al.*[19] and relabelled. **d)** is reproduced with permission from Figure ten in [23] and partially relabelled. R.O. Weller (personal communication) has emphasized that the spaces shown between the arterial wall and the pial sheath and between the sheath and the glial end-feet were virtual spaces in their electron micrographs. The periarterial spaces are also portrayed in Figure 6.

The contents of the skull (Figure 1) consist of the following: the brain parenchyma comprising cells surrounded by ISF together with connective tissue; the vasculature; the meninges overlying the brain; and the CSF that fills the ventricles within the brain and the spaces on its outer surfaces. The ISF, blood plasma and CSF collectively make up the extracellular fluid of the brain and spinal cord. Working inwards from the skull (Figure 1c), there is a thick supporting layer of tissue (the dura), another connective tissue layer (the arachnoid membrane), the pia mater and the brain parenchyma with its glial outer surface. The arachnoid membrane and pia mater are separated by the subarachnoid space that is bridged by chordae and larger trabeculae (Figure 1d). The space between the pia mater and the parenchyma, called the subpial space or space of His, may be a virtual space, i.e. with zero thickness [1, 22]. The parenchyma is made up of cells, e.g. neurons and glia, the extracellular matrix and ISF that pervades the matrix and fills the spaces between the cells. Arteries and veins run through the subarachnoid spaces with branches penetrating into the parenchyma [23]. Veins leaving the parenchyma drain into the various venous sinuses and thence to the jugular veins.

When considering fluid balance, the brain can be thought of as a greatly distorted tube, sealed at one end. Starting from the blind end, the spaces inside this tube include (Figure 1a), a pair of lateral ventricles, one in each cortical hemisphere, connected to a midline third ventricle, which in turn is connected via the cerebral aqueduct (or aqueduct of Sylvius) to a midline fourth ventricle that leads to both the central spinal canal and, via foramina of Magendie and Luschka, to the subarachnoid spaces outside the tube. All of these spaces including the central canal and the subarachnoid space of the spinal cord are filled with CSF. Because the skull is a rigid box, the sum of the volumes occupied by the parenchyma with its ISF and associated connective tissue, the vasculature, the meninges and the CSF must be constant, a concept known as the Monro-Kellie dogma [24] which has implications for CSF movements during the cardiac cycle, see below. The spinal cord has the same components but less rigid constraints on its total volume [6].

### 1.3 Functions of the fluids

The ISF provides the environment within which the brain cells survive and function. Water and solutes enter and leave the cells from and to this fluid. As such its composition and volume must be controlled to allow reproducible and reliable cell activity. There must be mechanisms for rapid delivery of both oxygen and nutrients, especially glucose and possibly lactate but also amino acids and other substances. There must also be mechanisms for rapid removal of the principal waste products, especially carbon dioxide and possibly lactate. These requirements are met by having microvessels within the parenchyma sufficiently close to all cells that diffusion distances for these solutes are small. Regulation of Na^+^, K^+^, Cl^−^, Ca^2+^, Mg^2+^,  and other ion concentrations in the ISF is important for correct nervous activity and regulation of the sum total of the solute concentrations, the osmolality of ISF, is crucial for regulation of cell volume.

The CSF serves a number of functions which include providing a) partial buoyancy for the brain, cushioning it within its rigid box, b) a means to compensate for the changes in blood volume within the skull during the cardiac cycle and c) a route for removal of waste products from the brain, particularly those of high molecular weight. CSF is also important in removing protein and other debris during recovery from injury or microhemorrhage. It may also serve as a route for dispersal of certain nutrients and hormones and other molecular signals which may be particularly important in brain development [25].

### 1.4 Composition of fluids

CSF and ISF are very similar in composition. K^+^, Ca^2+^, Na^+^ and Cl^−^ concentrations in ISF have been determined directly using ion selective microelectrodes [26, 27]. They have also been inferred from the modest changes in composition of CSF measured as it flows over the surface of the brain exchanging components with ISF [6, 9, 28, 29]. The composition of ISF and of CSF does, however, differ significantly from that of blood plasma (Table 1). This is because many hydrophilic substances are effectively prevented from entering the brain from the blood and many substances, including neurotransmitters, are greatly hindered in exiting from the brain (e.g. [6, 30]). The barriers preventing these movements include the blood-brain barrier separating blood and ISF within the parenchyma and the blood-CSF barriers comprising the choroid plexuses and the arachnoid and dura that surround the brain (Figure 1).

**Table 1:** **Comparison of composition of CSF and blood plasma**

Component	Ref.	CSF	Blood plasma
		Concentration/mg (100 ml)^-1^	
Protein	a	16 to 38	6300 to 8500
Sugar	a	45 to 80	80 to 120
Amino acids	a	1.5 to 3	4.5 to 9
Creatinine	a	0.5 to 2.2	0.7 to 2
Uric acid	a	0.4 to 2.8	2.9 to 6.9
Urea	a	5 to 39	22 to 42
Cholesterol	a	trace	100 to 150
Lactic acid	a	8 to 25	10 to 32
Phosphate (inorganic)	b	3.4	4.7
		Concentration/mmol kg^-1^	
Na+	b	147	150
K+	b	2.86	4.63
Ca2+	b	1.14	2.35
Mg2+	b	1.1	0.8
Cl^−^	b	113	99
	b	23.3	26.8
pH	b	7.3	7.4

Values are taken from the compilations in a) Millen and Woollam (1962) [2] based on data from Merritt and Fremont-Smith (1937) [31] and b) Davson and Segal (1996) [9].

### 1.5 Anatomical basis of barriers and routes of fluid transfer

**Figure 2:**
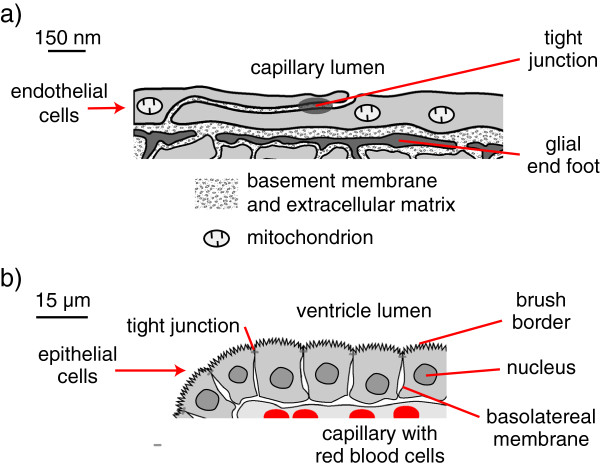
**Key features of a) blood-brain barrier and b) choroid plexus. a)** Sketch based on electron micrographs [34] of brain microvessel walls showing endothelial cells linked by tight junctions. These cells form a complete lining of the microvessel lumen and are in turn surrounded by glial end-feet. Occasional pericytes embedded in the basement membrane that separates the endothelial cells and the end-feet are not shown. The tight junctions limit the transport of solutes between the endothelial cells but the basement membrane, the gaps between the glial end-feet and the extracellular spaces within the parenchyma allow passage of molecules as big as proteins. Mitochondria occupy up to 10 % of the endothelial cell volume [35] and provide a substantial source of energy for transport. **b)** Diagram of one side of a choroid plexus villus as seen in light micrographs (e.g. [2]). Note the 100-fold difference in scales. Each choroid plexus has many such villi, each consisting of a layer of epithelial cells linked by tight junctions and surrounding a core of connective tissue containing capillaries. The endothelial cell layer lining these capillaries is not sealed by tight junctions. The epithelial cells have a prominent apical brush border facing the ventricle and a band of tight junctions separating the apical and basolateral domains of the cell membrane. There is folding of the basolateral membranes, which like the individual microvilli of the brush border are not visible in light micrographs (for electron micrographs see Figures two & twelve in [15]). The microvilli and basolateral folds increase the surface area of the membrane domains and hence the number of transporters that can be located on the two sides of the cells. Choroid plexus epithelium has the structure and properties of a leaky epithelium capable of transporting large quantities of isosmotic fluid.

The blood-brain barrier (Figure 2a) is formed primarily by the layer of endothelial cells lining the microvessels within the brain parenchyma [32, 33]. These cells are joined to each other via tight junctions that effectively block free passage of molecules and ions between cells across the endothelial layer, i.e. the paracellular route. Substances required to enter or leave the brain across the barrier must of necessity be transferred through the cells. They must either pass through the lipid portions of the plasma membranes, if they are not too hydrophilic, or be transferred via specific transporters (e.g. GLUT1 for glucose) located on each side of the cells.

The CSF-containing spaces are bounded by: the arachnoid membrane on the outside surface of the subarachnoid spaces, the pia mater on the inner surface of those spaces, the ependyma lining the ventricles and the choroid plexuses. The arachnoid membrane consists of one or more layers of cells connected by tight junctions [36] and these layers are presumed to be the basis for the impermeability of the arachnoid membrane (see [9] for further discussion). The choroid plexuses (Figure 2b) consist of a layer of epithelial cells facing ventricular CSF and overlying permeable capillaries of the fenestrated type. The barrier separating CSF from blood is provided by the epithelial layer rather than the underlying capillary endothelium [37]. The cells of the choroidal epithelium are sealed together by tight junctions that limit paracellular movement of most materials between CSF and peripheral circulation [20].

**Figure 3:**
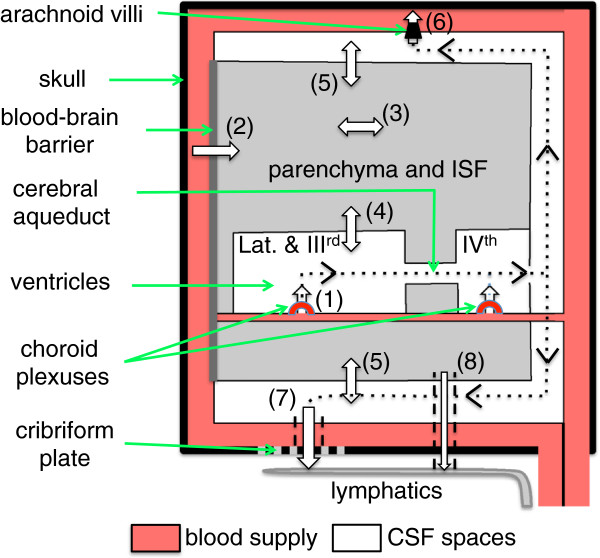
**Schematic plan of the whole brain indicating the fluid movements considered in this review.** Fluid is secreted into the ventricles across the choroid plexuses (1) and into the brain parenchyma across the blood-brain barrier (2). Fluid components can move through the parenchyma (3) and there are exchanges of water and solutes (4) and (5) between the interstitial fluid (ISF) of the parenchyma and cerebrospinal fluid (CSF) contained in the ventricles and in the subarachnoid spaces respectively. There is net fluid outflow across the arachnoid villi (6) leading to the dural venous sinuses (including but not restricted to the superior sagittal sinus) and along cranial nerves, most notably the olfactory nerve leading to the cribriform plate (7) and thence to the nasal mucosa. There may also be outflow of fluid in the walls of arteries or veins (8) leading to lymph nodes in the neck. The traditional view of the directions of net CSF flow is indicated by the dotted lines with arrowheads.

Small solutes appear to move freely across the boundaries between ISF and CSF. The inner surfaces of the parenchyma facing the ventricles (Figure 1b) are lined with ependymal cells, which do not appear to possess tight junctions that would provide a barrier such that even substances as large as inulin can pass between them [12, 32, 38–40]. Nevertheless loss of inulin or albumin from ventricular CSF into ISF is slow (at least in the absence of oedema), simply because diffusion of large molecules is slow and there is almost no convection. The outer surfaces of the brain parenchyma, defined by the glia limitans and the pia mater (Figure 1c), also do not provide a barrier to the movement of water and small solutes (section 4.1.1.1). Albumin injected into the parenchyma is slowly cleared from the ISF: some of it reaching CSF and some transported back to blood via other routes (section 4.1). The interfaces and routes of transfer important in production, circulation and removal of ISF and CSF are indicated schematically in Figure 3. An overriding principle is that what goes in must come out. An important way to address the rates of formation of CSF and ISF is to measure their rates of removal and this has been the subject of much investigation.

There is abundant evidence that much, probably most, of the CSF is formed by secretion by the choroid plexuses that are located in the brain ventricles, but the importance of ISF from the brain parenchyma as a source of CSF remains controversial. The principal sites at which CSF leaves the CNS are currently thought to include [16] the arachnoid villi linking cranial subarachnoid spaces to low pressure venous sinuses, the cribriform plate through which pass the olfactory nerves, and arachnoid villi located at the origins of the spinal nerves (Figure 1a).

## 2 Basic principles of fluid movements in the brain and lessons from studies on peripheral tissues

In gaining a better understanding of how ISF and CSF are formed and removed, it is necessary to consider a number of basic principles: not least, the distinctions between *diffusion* and *bulk flow/convection* and between *filtration* and *secretion*. Some aspects of these issues particularly as they apply to drug delivery to the brain have been discussed recently by Wolak and Thorne [41].

### 2.1 Diffusion

Diffusion is the observable movement of substances resulting from their random thermal motions. For a neutral solute in a stationary fluid, diffusion occurs down a concentration gradient. For ions, diffusion occurs down the resultant of the concentration and electrical gradients. For solutes crossing a lipid membrane, diffusion is best thought of as occurring down the gradient of electrochemical potential, defined in thermodynamics to include effects of differences in concentration, pressure and electrical potential. Diffusion occurs because all molecules are constantly in motion colliding and exchanging energy with their neighbours. For a neutral solute in a fluid, these random movements can generate net movement of the solute simply because there will be more solute molecules moving away from regions with high concentrations than there will be solute molecules moving away from regions with low concentrations. In a dilute solution, the movement of each solute molecule is independent of the movements of the others: there is no co-operativity. Because more massive particles have lower velocities, the rate of diffusion decreases as the mass of the solute increases. Diffusion is very fast over very short, atomic distances but because it is a random walk of short jumps with frequent changes of direction it is very slow over large distances.

### 2.2 Bulk flow or convection

Bulk flow, otherwise termed convection, is the movement of a fluid where all components of the fluid tend to move together. For example in a river the water and all it carries moves by bulk flow. In pure bulk flow, movements against the flow can be ignored. Furthermore all components irrespective of their size within a small volume of the fluid, providing it is sufficiently far from all boundaries, will move at the same speed.

### 2.3 Instances and relative importance of diffusion and bulk flow

When considering transport through extracellular fluid, cytoplasm or even the interior of a lipid bilayer, diffusion is the dominant process over short distances, i.e. less than a few microns and sometimes up to a few millimeters provided a concentration gradient exists. Bulk flow is the dominant process when transfers occur over long distances. Examples of diffusive movements include transfer of oxygen across a cell membrane and transfer of water across a lipid bilayer [42]. An example of bulk flow is blood flowing through the vasculature.

In most instances the actual process is neither just diffusion nor just bulk flow but something intermediate. A single process is actually occurring but it is often convenient to describe it as the sum of a movement due to diffusion superimposed on a movement due to flow of the fluid as a whole. Partly to emphasize that movements against the flow cannot be ignored, the flow component is often called convection rather than bulk flow.

Though convection is difficult to avoid in an unconfined space (even small differences in density resulting from differences in temperature are sufficient to produce eddies) large forces are required for flow to occur in confined spaces. This is illustrated by Poiseuille’s equation showing that the pressure difference required, Δ*P*, to produce a flow, *Q*_*V*_, through a tube with internal radius, *r*, increases with the inverse fourth power of the radius,


It is this strong dependence on the size of the spaces available for flow which accounts for the very high resistance to flow through the interstitial matrix normally found between cells, but the more than 1000-fold lower resistance to flow in the same tissues when the matrix is expanded by oedema [43–45]. The resistance to flow in peripheral tissues appears to be vested in the nature of the gel within the interstitial spaces rather than the sizes of the spaces themselves. It has been estimated that in synovium [45] which has an extracellular space of about 20 % (within the range associated with grey matter in the brain [9, 46]) the actual resistance is about 5000-fold greater than if the spaces were filled with free fluid.

### 2.4 Transfers by specific transporters

In addition to diffusion or convection, transfer across membranes can often involve interactions with specific transporters, which include channels (e.g. Kv1.3 for K^+^); cotransporters (e.g. NBCe1 for Na^+^ and ) and exchangers (e.g. AE2 for Cl^−^ and ). Cotransporters and exchangers are examples of carriers. A more complicated carrier, the Na^+^, K^+^-ATPase couples the energy released by hydrolysis of ATP to force Na^+^ and K^+^ to move against their gradients of concentration and electrical potential. For that reason this type of transporter is often called a pump. The Na^+^-pump is the key link between energy released from metabolism and the secretion processes in the choroid plexuses and at the blood-brain barrier.

### 2.5 Filtration, secretion and absorption

Net movement of fluid across barriers can be described as filtration or secretion. Either process in reverse is called absorption.

#### 2.5.1 Filtration

Filtration is the production of a net volume flow across a barrier driven by hydrostatic pressure and/or osmotic pressure gradients. The energy source for the movement of water and solutes comes from outside the barrier, not from the processes occurring within the barrier.

Filtration across the walls of capillaries is often described using the language of non-equilibrium or irreversible thermodynamics. In this the net volume flow per unit area, the volume flux, is given by:


where *L*_p_ is the hydraulic permeability, Δ*P* the hydrostatic pressure difference across the membrane, *R* the universal gas constant, *T* the absolute temperature, Δ*c*_*i*_ the concentration difference for the *i*th solute (e.g. Na^+^, Cl^−^, or serum albumin) and σ_i_ the corresponding reflection coefficient [47–49]. The reflection coefficient for a particular solute and a particular membrane is usually defined as the ratio of the osmotic pressure difference produced by that solute across that membrane to the osmotic pressure difference that would result if the solute were totally impermeant [47, 49]. The name “reflection coefficient” stems from its appearance in another equation from the same theory that applies when there are no solute gradients and the fluxes are driven solely by a hydrostatic pressure difference,


where *J*_*i*_ is the flux of the *i*th solute present on both sides at concentration *c*_*i*_. Thus if all of the solute is reflected σ_i_ = 1 and there is no flux of solute, while if none is reflected σ_i_ = 0 and the solute is transferred along with the water. One of the main results of the application of non-equilibrium thermodynamics to membrane transport is that the reflection coefficients are the same in the two equations given above [47, 48]. These equations come with two caveats: they have a firm basis only when the forces and flows are small enough that each flow is a linear function of the individual forces [50] and strictly the concentration differences should be differences in thermodynamic activities, which is important in quantitative work as the osmotic pressure of the proteins deviates markedly from the ideal value calculated from van’t Hoff’s law, Δ*π* = *RT*Δ*c*.

The Starling mechanism is a special case of filtration that accounts for fluid movement across the walls of peripheral capillaries. Starling [51] divided the solutes present in plasma and extracellular fluid at osmotically important concentrations into two groups: small molecules, the crystalloids like Na^+^, Cl^−^ and glucose, which can easily cross peripheral microvessel walls, and all the rest, the colloids, like serum albumin which cross very slowly. Using this distinction he proposed that fluid movement across microvessel walls can be described as net filtration or reabsorption of a fluid composed of water and crystalloids driven by the differences of the hydrostatic pressure and the osmotic pressure of the colloids. The latter has been called variously the colloid osmotic pressure or the oncotic pressure. Starling’s simplifications of the actual process cannot describe short-term transients when for instance the plasma NaCl concentration is abruptly changed but have otherwise been remarkably successful in describing fluid movements into and out of peripheral tissues (e.g. [49, 52, 53], see Endnote^a^).

In the language of non-equilibrium thermodynamics, Starling’s approximation amounts to saying that σ_*i*_ is 1 for the colloids and *σ*_*i*_Δ*c*_*i*_ approaches zero for all of the crystalloids. It has proven very difficult to determine the values of σ_*i*_ for small solutes and peripheral microvessels because permeation is so fast that equilibration between plasma and the interstitial fluid immediately outside the microvessels is reached during the measurement, i.e. Δ*c*_*i*_*≈ 0*[54]. The “true reflection coefficients” may be much greater (e.g. 0.5) than the “apparent reflection coefficients”, 0.01 to 0.1, often quoted (for discussion see e.g. [54–56]).

##### 2.5.1.1 Mechanistic descriptions of filtration

The treatment of filtration using irreversible thermodynamics assumes that the energy sources for filtration are the hydrostatic and concentration gradients. It makes no attempt to elucidate the actual mechanisms involved other than to stipulate that they must explain the values of *L*_*p*_ and σ_*i*_. Attempts at mechanistic descriptions have been complex and controversial and none has achieved widespread acceptance. For discussion of some of the difficulties see e.g. [53, 57, 58]. Bulat and Klarica [59], alongside their consideration of brain fluid movements, have proposed that in peripheral capillaries water moves down the resultant of the hydrostatic and total osmotic gradients and that the resulting change in total osmotic pressure rather than the colloid osmotic pressure within plasma limits the water fluxes and thus filtration. However, their proposal ignores the effect of Na^+^ and Cl^−^ fluxes across the peripheral capillary walls that reduce the local solute concentration differences. In its present state this description is not an improvement on the Starling approximation.

#### 2.5.2 Secretion and absorption

Secretion of fluid is a net volume flow into a region of interest, driven by energy derived from metabolism within a barrier (e.g. [5]). The energy is supplied as part of the transport of solutes; the water follows osmotically but see e.g. [60, 61] for consideration of the possibility of specific active water transport. Absorption or reabsorption of fluid could in principle be either filtration (including non-selective filtration, i.e. bulk flow) or secretion of fluid each directed back towards the blood. At least part of the absorption of CSF may be a non-selective flow of fluid carrying all its components through the arachnoid villi or the cribriform plate.

### 2.6 Uses of tracers or marker substances and lessons from the periphery

**Figure 4:**
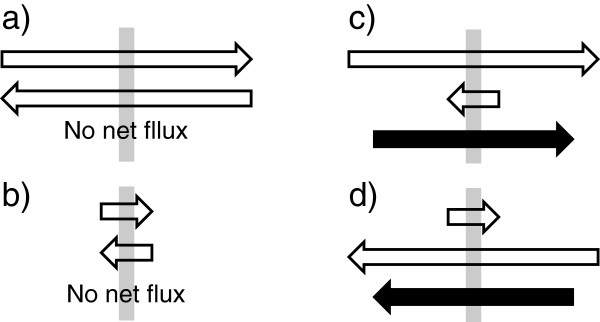
**Fluxes inwards or outwards across a membrane compared with net flux.** The membranes are indicated by the grey bars. In **a)** and **b)** the magnitudes of the fluxes are very different but in both cases the fluxes in the two directions are equal and hence there is no net flux. In **c)** and **d)** the magnitudes of the fluxes in the two directions are very different and there is a net flux in the direction indicated by the black arrow. Although the flux from left to right is the same in **a)** and **c)**, because the fluxes in the opposite direction are not the same there is a net flux in **c)** but not in **a)**. Hence net flux cannot be inferred from measurements of flux in a single direction.

Following the movement of tagged substances, i.e. tracers, has been a common method both for determining the characteristics of flow of the various brain fluids and for investigating transport processes across the choroid plexuses and the blood-brain barrier. The nature of the particular tracer used is crucial in the interpretation of results. For instance to measure flow, the marker must move only because there is net flow, must move at the same rate as the flow and must be easily detected quantitatively. Such markers have included inulin, dextrans and serum albumin. Other markers such as India ink particles and horseradish peroxidase have been very useful for tracing routes, but not for determining rates of flow. Tritiated water is not suitable for measuring rates of bulk flow because it is not constrained to stay within the fluid for which flow is being measured. (A well-known example is the flow of fluid through a capillary as described below). Furthermore, additions of tritiated water on just one or the other side of a barrier cannot be used to measure secretion or absorption because water moves so rapidly in both directions by diffusion. Net flow across a barrier occurs only if the fluxes of water and solutes in one direction exceed those in the other direction (Figure 4). It is possible for there to be large tracer fluxes while not having net flow. In that case the fluxes in the two directions are equal in magnitude.

To illustrate the importance of distinguishing the flux in one direction from net flux, one can consider the net flux of water across peripheral capillaries and venules of e.g. skin or muscle. The net flow can be determined by measuring water content, volume or weight changes in the tissues since almost all of the fluid is water. The result for non-fenestrated peripheral capillaries, which has stood the test of time, is that the net rate of filtration into the tissue is of the order of 0.1 % of the blood flow [62]. This filtration is driven by the Starling balance of forces, i.e. the net result of hydrostatic and colloid osmotic pressure differences (section 2.5.1). This net driving force can be expressed as a pressure difference or, using Δ*π* = *RT*Δ*c* with *RT* = 19 mmHg/mM, as the concentration or osmolality difference Δ*c* that would produce the same osmotic pressure, i.e. 19 mmHg is equivalent to 1 mM. The net driving force is very small, reduced by local concentration gradients in the regions of flow.

The water fluxes into and out of a tissue can be determined using radiotracers or heavy water. These fluxes are found to be between 1000- and 10000-fold greater than the net rate of filtration of water. Virtually all of the tritiated water molecules delivered by the blood cross the capillary wall before the blood leaves the tissue and thus transfer is limited by blood flow [55, 63, 64]. How is it that the net flux of tritiated water appears so large while the net flux of water is so small? The measurable net flux of tritiated water is driven by its own concentration gradient, which on a first pass through a tissue means effectively by its concentration in plasma since the concentration in the much larger volume of the tissue remains very small, i.e. the influx of tritiated water is almost equal to the net flux of tritiated water. Since, near enough, tritiated water and normal water behave the same, this means that influx of water, the inward unidirectional flux (Endnote^b^), is driven by its total concentration, approximately 55 M. However, the net flux of water, the filtration, which can be measured by volume change, is the difference between the influx and an almost equal efflux and thus it is driven by an equivalent concentration *difference* of water, which across a peripheral capillary wall is approximately 1 mM. Because the driving forces are so different, 55 M vs. 1 mM, it should not be surprising that the unidirectional flux is much larger than the net flux. Similar comparisons can be made for Na^+^[55, 63, 64]. The molecular mechanisms for movements of Na^+^ and water across the walls of peripheral microvessels look more like diffusion than flow, but nevertheless the net process is still filtration. The traditional [52] and current [49, 53] views on filtration and reabsorption in peripheral capillaries are described briefly in Endnote^c^.

It is important to keep in mind that the terms filtration or reabsorption each describe a net process. They do not describe the movements in a single direction. No one now describes the process of fluid movement in peripheral capillaries as filtration of 99.9 % into the tissue with reabsorption of 99.8 % leaving 0.1 % in the tissue to be removed in the lymph flow. The difficulty is not with the numbers, as these are approximately the proportions of water molecules that move, but with the use of the words. These points are clear in the application of the principles to microvessels in the periphery. However, there are repeated instances of reports concerning fluid movements in the brain that have not been careful to distinguish between tracer fluxes and net fluxes of water and hence have incorrectly identified rates of secretion, filtration, absorption or flow of CSF and ISF (e.g. [65–72]).

### 2.7 Application of basic principles to blood vessels in the brain

**Figure 5:**
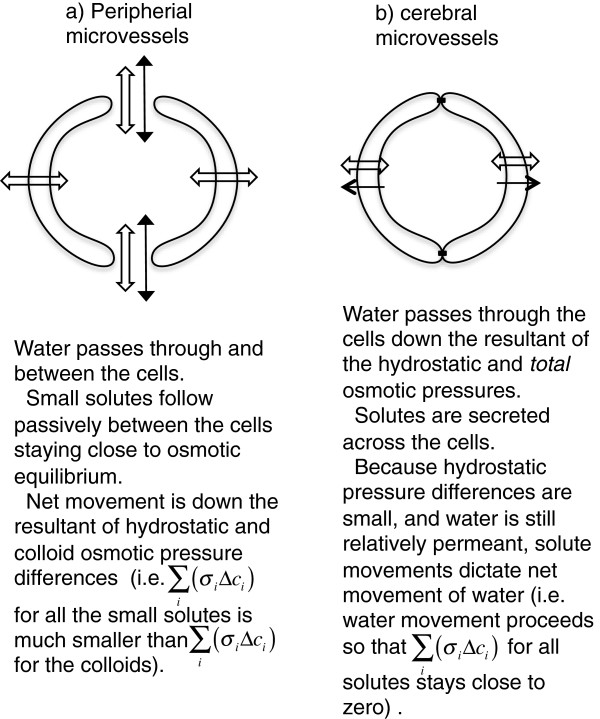
**Comparisons of fluid transport across peripheral and cerebral microvessels.** In the periphery **a)** small solutes cross the vessel walls via gaps between the cells. Small solute movement is rapid, therefore their concentration gradients are small and thus unlike the large solutes, the colloids, the small solutes do not oppose the movements of water. Thus except during brief transients, e.g. when osmotic gradients are artificially imposed, net fluid movement is governed by the hydrostatic and colloid osmotic pressure differences between blood and surrounding tissues. In the brain **b)** paracellular movement is limited by tight junctions. Thus small solutes cross the vessel walls only slowly and the direction and extent of their movement is determined by specific transporters. Hence they are as effective as the large solutes in producing osmotic gradients that dictate the extent of water movement into or out of the surrounding tissue.

The same physical chemistry applies to brain microvessels as to peripheral capillaries, but there are important differences in the barrier properties of the vessel walls and in the transport pathways across them (Figure 5). In the periphery Na^+^ and Cl^−^ cross the capillary walls easily in both directions and except possibly during relatively brief transients, e.g. when osmotic gradients are artificially imposed, their concentrations in the filtrate are similar to those in plasma. By contrast, at the blood-brain barrier, the low resistance pathways shared by water and hydrophilic solutes are absent, i.e. the paracellular route is blocked by tight-junctions. The blood-brain barrier has at least a 10 fold lower permeability to net flow of water than peripheral capillaries but the fluxes of water are still large enough that ca. 90 % of tracer water is extracted from the blood in a single pass [73–75]. Thus movements of water can occur relatively easily when gradients of total osmolality are imposed. Osmotic equilibrium is approached on a time scale of a few minutes [76–78] but equilibration half-times measured at various locations within the brain vary, presumably reflecting differences in the ratio of the surface area of microvessels to the local tissue volume [78]. Osmotically driven movement of water is the basis of the clinical use of hyperosmotic mannitol in plasma to draw water out of the brain to relieve elevated intracranial pressure. Net water movements in response to imposed osmotic differences are properly called filtration or absorption depending on the direction. They can be described (as in section 2.5.1) as flow driven by the resultant of hydrostatic and *total* osmotic pressures. This is not the same as the Starling mechanism concerned with fluxes of water and all small solutes across peripheral membranes. Starling’s approximations only work when small solutes permeate rapidly (section 2.5) and this is not the case for the blood-brain barrier.

The permeability of brain microvessels to Na^+^ and Cl^−^ is more than 1000-fold lower than that of peripheral microvessels and the reflection coefficients of these and many other small solutes, e.g. sucrose and raffinose, are indistinguishable from 1 [7, 73]. This has very important implications for the “normal” movements of water and solutes in the absence of imposed osmotic differences. Any filtration driven by a difference in hydrostatic pressure will contain very little solute (section 2.5.1). Because even a small, e.g. 1 mM, concentration difference for NaCl across the barrier represents the same driving force as an unphysiologically large, e.g. 38 mmHg (= 56 cmH_2_O), hydrostatic pressure difference, the rates of water and solute transport must be closely matched. Any possible increase in hydrostatic pressure difference will only produce a small flow of water that becomes self-limiting because it will reduce the osmolality in the interstitium. Furthermore net reabsorption of fluid driven by hydrostatic or colloid osmotic pressure gradients must leave the solutes behind and thus the reabsorption will be self-limiting. It can at most be very small compared with the rates at which CSF is produced.

Viewed the other way round, net solute transport across the blood-brain barrier will be matched by water flow. In other words, water follows salt. One could say that solutes are actively transported across the blood-brain barrier and water follows by filtration but the convention is to refer to the entire process as secretion of fluid. This has the important advantage that it emphasizes that the energy driving the process is derived from metabolism within the blood-brain barrier not from applied pressure gradients. *The rate of secretion of fluid across the blood-brain barrier is determined by the net rate of solute transport.* Furthermore, so long as water can move sufficiently rapidly between plasma and ISF that the osmolalities of these fluids are approximately equal, *the volume of ISF in the brain is determined by the solute content of the ISF and the osmolality of the plasma.*

Reabsorption across the brain microvessels of parenchymal fluid derived from the CSF has been proposed repeatedly [11, 65–70, 79–84] but in no instance have these proposals included a mechanism for reabsorption of the solutes. As explained above, the net pressures that can be produced, colloid osmotic or hydrostatic cannot drive net removal of fluid across the intact blood-brain barrier at a rate capable of dealing with excess fluid brought into the brain parenchyma (section 4.2.2 and Endnote^d^). This is not a new idea; this inability of the intact blood-brain barrier to reabsorb interstitial fluid has been argued before by many others [5, 7, 85–87]. Reabsorption could occur if the blood-brain barrier were to become permeable to small solutes, but this would amount to barrier failure and would mean it could no longer control ISF composition. The reason that removal of fluid across the intact blood-brain barrier cannot match the rate at which fluid is produced is that there is no mechanism for *net* transport of NaCl across the barrier in the direction from brain to blood. If salt could be transported, then water would follow.

The rate of secretion of Na^+^, Cl^−^ and  is the primary determinant of the rate of fluid secretion into the parenchyma. Metabolically-produced water does not affect these rates and hence has little impact on fluid secretion [88]. The water derived from metabolism will either be absorbed across the blood-brain barrier or reduce the amount of water needed to accompany the secretion of solutes by the blood-brain barrier.

### 2.8 Diffusion and convection within the parenchyma

By analogy with peripheral tissues (section 2.3), the expectation is that movements of water and solutes in brain ISF will be primarily by diffusion rather than convection. Thus diffusion is the mechanism for movement of molecules between adjacent cells. On the distance scale of capillary to parenchymal cell for which estimates range from 10 μm to 50 μm [89–92] the dominant process still clearly is diffusion and this accounts for the important movements of O_2_, CO_2_, glucose and lactate. The sole exception may be local circulation of K^+^ accompanied by water, a story tied up with the idea of glial K^+^ buffering which is beyond the remit of this review. For distances up to a millimeter or more, diffusion still appears to be the most important process but additionally there is convection along fibre tracts in white matter [93] and other privileged pathways, which may include capillary basement membranes and the perivascular spaces along arteries and veins. These privileged routes will be considered in section 4. The evidence that diffusion is the dominant process within grey matter is considered immediately below. More extensive reviews of this subject and related matters are available [41, 46, 94–96].

The early studies on transport within the extracellular spaces of the parenchyma were aimed primarily at demonstrating that these spaces exist, a hot issue in the early 1960s. These studies were based on measured concentration profiles within the parenchyma of radiolabelled solutes chosen for their inability to enter cells or cross the blood-brain barrier [97, 98]. At various times after the labelled solutes were added to the ventricles (and kept at constant concentration by perfusion), the animals were killed, and parenchymal samples were taken at known distances from the ventricular surface. The concentration of solute found in the tissue adjacent to the perfused surface provided a measure of the volume occupied by the extracellular space while deeper samples provided information about diffusion. These values were then compared with those expected theoretically if transport were to be by diffusion:


where *C*(*x*) is the concentration at distance *x*, *C*_0_ is the concentration at the ventricular surface, *t* is the time after addition of the solute, *D* is the effective diffusion coefficient and erfc is the complementary error function. Hindrance to diffusion imposed by the tissue is described by the tortuosity, defined as the square root of the ratio *D*_0_/*D*, where *D*_0_ is the diffusion coefficient in a free medium. The comparison of experiment and theory provides two tests of whether or not solute movement is by diffusion by asking: “Do the data fit the shape of the theoretical curve?” and “Is the value calculated for the diffusion constant the same for different times?” It was found that the data obtained by this method pass both of these tests. However, Rall [38] has stated quoting C.S. Patlak that these tests could still be passed even if there were convection with a flow towards and across the ependyma of up to 1/4 of the CSF production into the ventricles. In other words the data do not exclude convection that has been inferred from experiments on efflux of test substances from the parenchyma (section 4.1).

More recent determinations of transport through interstitial spaces have been based on injection or iontophoresis of a test substance at a point in the parenchyma and detection in real-time of its spread through the tissue. In the Real Time Iontophoresis method using tetramethylammonium ions (TMA), these cations are iontophoresed from a microelectrode and their concentration measured a known distance away with an ion-selective microelectrode [95, 99]. This technique can be calibrated to give actual concentrations resulting from known amounts of TMA added in a very small region and enables both volume fraction and tortuosity to be calculated. In the Integrative Optical Imaging method, a fluorescent marker is pressure injected and the concentration profile determined from microscopic observation of the fluorescence as a function of position in the surroundings. This is more difficult to calibrate and only yields a measure of the effective diffusion coefficient and tortuosity for the marker, but it has the advantage that it allows checks on the symmetry of the spread away from the injection site [46, 95, 100]. For instance it can show that spread in white matter occurs preferentially parallel to the long fibres. The large amounts of data obtained using these techniques are consistent with diffusion as the mechanism of transport over short distances. Flow velocities around 10 μm min^-1^, as inferred from tracer studies of solute flux (section 4.1 and [93]) would be too small to change significantly the calculated values of the diffusion parameters (Charles Nicholson and Tony Gardner-Medwin, personal communications) and even the larger flows inferred from some more recent measurements (see caveats in section 4.1.1) may be difficult to detect.

Convection may be more important as a mechanism for fluid movement in the brain under conditions where there is oedema associated with expanded extracellular spaces as can occur in white matter. By analogy with peripheral tissues, the resistance to convection may then be much smaller than in non-oedematous tissue (section 2.3). Reulen *et al.*[101–103] have considered convection in some detail in connection with the spread and resolution of oedema. Convection is important in the spread of drugs injected into the parenchyma in comparatively large volumes, so called convection-enhanced drug delivery [104–106] although the extent to which the flow passes through the interstitial spaces between cells in grey matter rather than being restricted to privileged pathways such as perivascular spaces has apparently not been determined.

### 2.9 Secretion by the choroid plexuses and the blood-brain barrier

The reader interested in the now historical argument about whether transport across the choroid plexus is secretion or filtration should consult reviews by Cserr [4], Davson and Segal [9] and Damkier *et al.*[15]. As discussed by Damkier *et al.*[15] the choroid plexus has all the hallmarks of a high capacity secretory epithelium whose product is isosmotic with plasma. The blood-brain barrier is a barrier at least partly because the endothelial cells lining the microvessels lack the low resistance paracellular pathways for small solutes that characterize microvessels in peripheral tissues (Figure 5). Instead the endothelial cells are sealed together by tight junctions that prevent (or at least greatly reduce) the passage of markers like horseradish peroxidase and lanthanum [32, 34] and raise the electrical resistance to levels similar to those in tight epithelia [107, 108]. As discussed in section 2.7, this low paracellular permeability means that sustained net movement of fluid across the blood-brain barrier requires transport of solutes to generate an osmotic gradient. Water then follows, partly by simple diffusion across the membranes of the endothelial cells, but possibly also via various transporters [61, 109]. The mechanisms of ion transport at the blood-brain barrier have been reviewed by O’Donnell [18] and will be considered further in a sequel to this review.

## 3 Formation and removal of CSF and ISF

### 3.1 CSF

In 1996 when Davson and Segal published their monumental review [9] the general view was that the rate of production of CSF was roughly 400 ml/day in humans and that of this perhaps 90 % arose as secretion by the choroid plexuses and 10 % by secretion of ISF across the blood-brain barrier and subsequent flow to join CSF. However, both the underlying assumption that all ISF flows into the CSF and the relative contribution of ISF to the secreted CSF have been and remain controversial.

#### 3.1.1 Formation of CSF

If the choroid plexuses produce almost all the CSF, their removal should prevent the excessive fluid accumulation in the ventricles that occurs in hydrocephalus (section 4.2). Yet Milhorat [110–112] has stated quite firmly that choroidectomy does not work for the treatment of hydrocephalus and thus there must be sources of CSF in addition to the choroid plexuses. However, at least in the hands of Scarff [113], choroidectomy was found to be an effective therapy in a large proportion of patients with communicating hydrocephalus. One possible explanation of these discrepancies may be the extent to which total choroidectomy had been achieved. Regardless of the eventual resolution of the dispute over clinical utility, taking Milhorat’s and Scarff’s work together leaves little doubt of the basic physiological point, i.e. removal of the choroid plexuses reduces the rate of production of CSF but to nowhere near zero. The extensive series of choroid plexus cauterization treatments for hydrocephalus in children by Warf [114, 115] (see also [116]) is consistent with the choroid plexuses being a major, but not sole source of CSF even in children under one year (compare section 4.2.3).

#### 3.1.2 Removal of CSF

As reviewed by Pollay [16, 117], CSF is removed by several routes including the arachnoid villi leading to venous sinuses, perineural pathways across the cribriform plate leading to nasal mucosa, and pathways at the roots of spinal nerves leading either to blood or lymph [8, 9, 118–127]. Large solutes like albumin that are removed from the brain via the cribriform plate enter lymph. By contrast, a large proportion of the lower molecular weight solutes and water leaving by the same route reach the blood directly across peripheral capillary walls in the nasal mucosa [120]. The relative rates at which CSF is removed by the various routes may vary markedly between species.

#### 3.1.3 Measurement of CSF production rate

CSF production rate has been measured in several ways [9] of which three will be discussed here. These are a) collecting CSF as fast as it is formed; b) recording the rate of removal of injected markers into CSF; and c) measuring the dilution of markers perfused into the ventricles at a known rate. In the collection method a), CSF is removed through tubing or a needle held at a pressure sufficiently low that CSF exits solely by this route, it being assumed that all normal exit routes require a higher pressure. This was the method used in the influential study by Ekstedt [128, 129]. **Caveat:** It is no longer clear that this procedure can prevent exit of fluid from the brain by other routes. There may be large variations in the rate at which substances reach differing parts of the subarachnoid space when infused into the ventricles [130, 131]. Furthermore as discussed in section 4.1, it appears that some of the fluid secreted into the ISF in the forebrain returns to blood or lymph by routes that avoid mixing with CSF in the cisterna magna indicating that not all regions of the subarachnoid spaces are in free communication [119, 132]. These observations suggest that reducing pressure at the point of collection, e.g. cisterna magna or lumbar sac, may lead to collapse of part of the subarachnoid space and thus failure to transmit the reduction in pressure to other parts. Collecting CSF from some portions of the subarachnoid space may be a bit like trying to suck fluid through a flimsy straw. In the marker removal method b), a marker substance is injected into the cisterna magna and a complete collection of CSF is made at some later time. The rate of loss of the marker and the CSF production rate are calculated from the amount remaining. The calculation requires the assumption that CSF is well mixed throughout the period before collection and that all of the CSF can be collected. The method would still work if a portion of the CSF e.g. that in the spinal cord, were completely stagnant and were not reached by the marker and were not collected. **Caveat:** Studies such as those discussed in sections 4.1 and 4.3 indicate that CSF is not well mixed as required for these calculations. In the perfusion method c), fluid containing a known concentration of a marker is infused at a constant rate into a lateral ventricle and fluid is sampled by withdrawal from either the cisterna magna or lumbar sac. Assuming that the marker only leaves the CSF as part of fluid with the same concentration as in the sample, i.e. by bulk flow of well mixed CSF, and all of the secreted CSF is added to the mixture that is sampled, the secretion rate can be calculated as follows [133]:


where each *C* is the concentration in the indicated fluid. Note that this method does not require total collection of CSF and thus the sample can be small and can, at least in principle, be taken without reducing intracranial pressure. **Caveats:** If CSF were secreted solely from blood into the ventricles from which it flowed into the cisterna magna and was then reabsorbed into blood or lymph from the subarachnoid spaces, the assumptions needed for this calculation to be correct would be plausible. However, as will be discussed in section 4, these are not safe assumptions. CSF is not well mixed and, as a result, the marker concentration in the sample does not accurately reflect those in the various outflows. At present there can be no “gold standard” method for measuring CSF production at least partly because there is no single compelling definition of what CSF production means, e.g. rate of fluid secretion into the ventricles or alternatively rate of fluid secretion into the CNS as a whole. The present estimates are likely to be reasonable values for the normal rate of secretion of fluid into the ventricles but may be underestimates of the total rate of fluid secretion into the brain as discussed in the following sections.

### 3.2 Measurement of ISF production rate

Estimates of the rate of ISF production can at least in principle be obtained from measurements of a) the rate of CSF production after destruction or removal of the choroid plexuses [134, 135], b) the rate of entry of fluid into the perfused cerebral aqueduct [136, 137] and most convincingly c) the rate of removal of markers injected into the parenchyma [132, 138, 139]. **Caveats:** There are drawbacks to each of these approaches. As Milhorat [112] was careful to emphasize, the first a) requires total removal of the choroid plexuses (or evidence about the contribution of the portions remaining) and measures a rate of fluid production after the fluid dynamics have been severely altered. The second method b) requires the assumption that ISF production in regions that drain towards the aqueduct is typical of production in all regions. In addition, the measurements are made after extensive recent surgical intervention. The third method c) requires that measurements be made in a number of locations (cf. [139, 140]) and assumes that the tracer substance is carried out of the parenchyma by convection, that there is no sieving of the markers and that the flow arises only by secretion of fluid across the blood-brain barrier.

Sieving refers to the retardation of movement for large solutes when fluid flows through a filter, a matrix or a gel. Flow through a matrix does not lead to flow of solutes of all sizes at the same rate, a defining characteristic of pure bulk flow, unless the sizes are all well below the typical distance between the fibres of the matrix [141]. The nature of the matrix, if any, along the rate limiting portions of the flow pathway for drainage of interstitial fluid (see section 4.1) is unknown. Thus estimating fluid flow rate from the rate at which large markers are removed from the tissue may underestimate the net rate of flow of water and small solutes. The evidence for bulk flow and for the absence of sieving in the parenchyma for solutes smaller than albumin is that the measured rates of removal of tracers of a range of sizes up to albumin were all the same [132]. However, the rate limiting stage of the flow was held to be along privileged pathways including perivascular (or Virchow-Robin) spaces, spaces between fibre tracts in white matter and spaces within the subependymal layer of the ventricles [7, 138]. The evidence obtained from removal of large markers is considered further in section 4.1 and the assumption that flow through the parenchyma arises only from the blood-brain barrier is considered in sections 4.3 and 5.

## 4 Ongoing approaches to the investigation of brain fluid dynamics

### 4.1 Studies of movement of substances and routes of outflow from the brain parenchyma

To discover whether the production of ISF contributes to the CSF, it is necessary to find out whether ISF drains into CSF before leaving the brain or whether it goes via some independent route. As described below, drainage of ISF appears to be partly to CSF as sampled in the cisterna magna and partly to cervical lymph nodes by routes that do not require the emerging ISF to mix with CSF in the cisterna magna.

#### 4.1.1 Periarterial spaces as routes of efflux

When markers such as radiolabelled serum albumin or polyethylene glycols are injected into the caudate nucleus of one side of the forebrain, twice as much appears in the ipsilateral cervical lymph node as in the contralateral node in the neck, and less than 30 % reaches the cisterna magna [119, 132]. By contrast, if the same markers are injected into ventricular CSF a smaller proportion appears in the cervical lymph nodes but that which does so appears symmetrically on the two sides. These results have since been extended to other sites of injection revealing that the fraction of tracer reaching the cisterna magna varies depending on distance from the injection site [139, 140]. Furthermore, in experiments following the clearance of radiolabeled albumin, tracer concentration was found to be much higher in the walls of dissected arteries supplying the region where the tracer had been injected than in the surrounding CSF. For the caudate nucleus and internal capsule this prominently included the circle of Willis. Similarly, when horseradish peroxidase was injected (in the large volume of 30 μl) into the midbrain of a rat, it could be detected an hour later in or on the walls of branches of the posterior cerebral artery [139]. These findings were confirmed and extended [140] with the additional observation that the delay between the disappearance of tracer from the site of injection and its appearance in lymph was longer when the tracer was injected into the parenchyma than into CSF. **Caveats:** Two features of these studies have been challenged: the overall rate of removal and the assumption that injection of marker does not disturb the routes of outflow being examined. These experiments were all performed using barbiturate anaesthesia, but it has subsequently been shown that the half-life for efflux of ^14^C-sucrose, which crosses the blood-brain barrier at a negligible rate, is about 4 h when the rats are awake or anaesthetized with ketamine and xylazine but about 25 h when they are anaesthetized with pentobarbital [142]. In ketamine/xylazine anaesthetized mice efflux of mannitol, another solute with low blood-brain barrier permeability, has a half-life similar to 4 h [143]: this confirms the result for sucrose. If the half-life for efflux of albumin is similarly faster in awake animals than in those anaesthetized with barbiturates, the estimates of the ISF production rate given by Cserr and co-workers [132, 138, 139] may need to be revised upwards, perhaps by as much as 6-fold (but see section 4.3 for alternative explanations for rapid removal of solutes). The rate of efflux of albumin clearly needs to be investigated further with attention being given to the state of anaesthesia.

Another serious concern is raised by the suggestion that the rates of infusion or volumes injected may have been sufficient to alter the mechanisms and routes of elimination. Iliff *et al.*[143] suggested that the observation of efflux along arteries might represent an artefact of “high local intraparenchymal pressure from the injection”. Pressure-induced flow is used in convection-enhanced drug delivery [105]. However, even the lower end of the infusion rates employed, 0.5 μl min^-1^ to 20 μl min^-1^, is 8-fold larger than the rate, 0.5 μl infused over 8 min, used by Szentistvanyi *et al.*[139]. Thus it is difficult to see how the infusions in the reported studies could have altered the efflux route from a putative physiological perivenous route (section 4.3) to the periarterial route observed.

Bradbury, Cserr and colleagues were of the opinion that flow out of the parenchyma occurred along periarterial spaces [144] and that where the arteries reached the subarachnoid space there was some form of barrier separating the periarterial and subarachnoid spaces. Of the structures described subsequently by Zhang *et al.*[23, 145] (Figure 1d) this barrier may be the sheath of pia mater enveloping the artery. Cserr and Patlak and Bradbury *et al.* incorporated this route of efflux into a general scheme in which ISF is secreted by the blood-brain barrier and then flows out of the parenchyma to CSF via periarterial spaces. The pial barrier delays but does not prevent passage of the albumin into CSF destined for the ipsilateral side of the cribriform plate and thus to the nasal mucosa [8, 119].

Further evidence supporting the existence of periarterial spaces as routes of efflux from the parenchyma has been provided using two-photon microscopy together with 3 kDa cascade blue dextran, viewing being through a cranial window [146]. Following injection into the parenchyma, the marker was detected in a space surrounding the arteries and in the extracellular matrix of the arterial smooth muscle layer but not along veins. The authors state (data not shown) that the rates of removal of fluorescence were similar for 3 kDa and 70 kDa markers but no removal was seen for a 2000 kDa marker. Fluorescence decreased more rapidly in the interstitium and periarterial space than in the smooth muscle layer. The authors suggest that this is because there are multiple routes for removal from the interstitium but only one for the smooth muscle layer. An alternative explanation for these observations may be that the dextran binds within the extracellular matrix of the smooth muscle layer.

##### 4.1.1.1 Evaluation of the proposal that periarterial spaces provide an efflux route for markers

**Figure 6:**
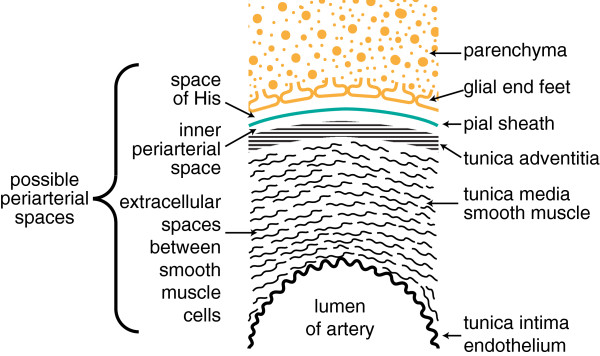
**Diagram indicating positions within the cortical parenchyma of periarterial spaces that may allow fluid movement.** The spaces shown correspond to those around the arteries in Figure 1d. The diagram has been formatted so that it may be compared with earlier published versions [1]. The diagram (not to scale) shows: the tunica intima, the endothelial lining of the lumen and a covering of elastic tissue; the tunica media, a smooth muscle layer; and the tunica adventitia, mainly connective tissue. Within these layers there are two possible free spaces. The inner, called the periarterial space by Zhang *et al.*[23] and labelled the inner periarterial space in the diagram, is continuous with the periarterial space of the subarachnoid portion of the same artery. The outer that in contact with the brain parenchyma, is likely to be the space described by His [1]. In the view of Weller and associates (personal communication) both the inner periarterial space and the space of His are virtual spaces with no thickness and fluid movement occurs preferentially in the extracellular spaces of the smooth muscle layer. Other interpretations of the spaces are considered in section 4.3.4.

The idea that markers can move from the parenchyma via convection in periarterial spaces (Figure 6) and thence transfer into CSF has a number of plausible features. The convection inherent in this idea would always occur along pathways that should have a low resistance to flow. Similarly, transfer to and from CSF across the pial barrier should be rapid for small solutes [97, 143, 147–153] (but see section 4.3.1). It is slow, but still easily demonstrable, for macromolecules [32, 138, 143, 154]. The actual permeabilities for transport between CSF and parenchyma across the pia mater are not known because concentrations on both sides of the barrier have not been measured simultaneously. Some of the large solutes emerging from the parenchyma not only reach CSF but also find their way to the cisterna magna [119, 132]. This scheme suggests a plausible explanation for the observation that the proportion of substance reaching the cisterna magna is larger from injection sites that are closer to the cisterna magna.

Rapid inflow via a periarterial space may also partly account for the early observation that horseradish peroxidase injected into CSF reaches the basement membranes of even the small microvessels in the cortex within a few minutes [79, 154–156] but at low concentration, such that at early times only very sensitive detection methods are capable of establishing its presence [157]. Rapid appearance of low concentrations deep in the cortex is the behaviour expected for relatively rapid transport over the large distance (millimeters) from the cortical surface, provided this is preceded or followed by slow penetration through a barrier. The rapid transport along the periarterial spaces could be provided by stirring or mixing in the perivascular spaces driven by the arterial pulse (compare [143, 154, 156, 158]). The barrier could be the pial coating of the arteries, which would precede the rapid transport process, or it might be the basement membranes or the layer of glial end-feet associated with the microvessels, either of which would follow the rapid process. There does not appear to be any data relating to the mechanism of transport for horseradish peroxidase through the subarachnoid space (section 4.3).

Whether veins also constitute an exit route for injected albumin from the parenchyma is not known. Most studies have not looked at this aspect, but in their fluorescent marker studies, Arbel-Ornath *et al.*[146] examined arteries and veins and saw fluorescence only along arteries.

**Caveats:** While efflux of solutes like albumin via periarterial spaces, CSF and the cribriform plate is an attractive proposal, it nevertheless raises a number of questions:

1. Can albumin be detected in sufficient concentrations near the cribriform plate for the nasal route to be able to account for the removal of the markers? Does obstruction of the cribriform plate reduce transfer of markers injected into the parenchyma to cervical lymph nodes as it does [120] for markers injected into CSF (see also [159, 160])?
2. Why have some markers (section 4.1.2) been difficult to detect in the periarterial spaces? The present lack of such data in images of brain sections (e.g. [161]) is perhaps to be expected for two reasons: as the spaces are thought to have rapidly changing dimensions they may collapse during sample preparation and the concentrations in these spaces during efflux of markers may be low (section 4.3 for further discussion).
3. Does the use of barbiturate anaesthesia alter the routes of efflux as well as reducing the rate (section 4.1.1)?

#### 4.1.2 Extracellular spaces of the arterial smooth muscle layer as routes of efflux

Weller, Carare and colleagues have been pursuing an alternative hypothesis that flow out of the parenchyma occurs through the smooth muscle layer of the arterial walls (Figure 6). They have shown that fluorescently tagged 3-kDa dextran, 40-kDa ovalbumin and amyloid-β [153, 161–163] injected into the parenchyma become diffusely spread and associated with both the basement membranes of capillaries and the extracellular spaces separating the smooth muscle cells in arterial walls. Five minutes after injection into the parenchyma they found markers associated with the basement membranes. For tissue fixed after 30 min, the markers were still present diffusely in the parenchyma and were also found in the smooth muscle layer, but not within capillary basement membranes. For fixation after 3 h (for the dextran) or 24 h (for all markers), fluorescence was seen only in punctate form near the outside edges of the walls of arteries associated with macrophages. These data were interpreted as supporting clearance of the markers from the interstitium via bulk flow through microvascular basement membranes and the extracellular spaces between the smooth muscle cells of arterial walls [161].

**Caveats:** It is clear that markers can reach the extracellular spaces of the smooth muscle layer of the arteries from the parenchyma, but it is not clear that there can be significant flow along these layers. However, it would be unwise to dismiss movement via the smooth muscle layer as physically impossible without evidence. The data raise a number of questions.

1. Why have the fluorescent markers investigated been concentrated in basement membranes and the smooth muscle layer? Is there some form of association or binding? Is there a sieving effect with retention of larger solutes with higher flow of water and smaller solutes than suggested by the movement of the markers?
2. Can there be exchange between extracellular spaces of the arterial smooth muscle layer and nearby periarterial spaces? If so, efflux from the parenchyma might be via the periarterial spaces with the marker able to gain access to the smooth muscle from the adjacent periarterial space.
3. Why are the fluorescent markers not found within the endothelial basement membranes of the arteries or within the outer basement membrane of the arterial wall [161, 164]?
4. Why does fluorescent ovalbumin disappear from the capillary basement membranes while there is still diffuse fluorescence observed in the surrounding parenchyma [161]?
5. What is the driving force for flow along arterial walls? Is it, as suggested, the arterial pulse changing compression in the wall [161, 165]? Is there any structural basis for the “valves” which appear to be needed to make compression drive movement preferentially in the outward direction [165, 166]?
6. Can movement of markers along arteries from parenchyma to lymph node be measured? How long does it take for them to reach the subarachnoid space and subsequently the cervical lymph nodes?
7. What fraction of an injected load of marker can be accounted for by that moving along arteries and reaching cervical lymph nodes?
8. Does deposition of amyloid-β along presumed flow pathways obstruct fluid movement via these same pathways? What happens to water and small solutes?
9. How in earlier experiments did radiolabelled human serum albumin injected into the parenchyma [119, 132, 139, 140] reach CSF in the cisterna magna (see earlier discussion)?

Even if the smooth muscle layer of arterial walls is not the main route for fluid transfer, it may still be important in clearance of amyloid-β and other proteins. This aspect has been the emphasis of recent work, e.g. [164, 167, 168]. It should be noted that the periarterial routes for transfer discussed in section 4.1.1 and in this section and indicated in Figure 6 are not mutually exclusive. It is possible that all pathways mediate transfer of fluid and solutes. It is also conceivable that net flow is in opposite directions in different spaces or even in both directions at different times within one. These possibilities are considered further in section 4.3.4.

### 4.2 Studies of CSF flow and the implications of the flow patterns for sites and rates of production and absorption of CSF and ISF

#### 4.2.1.1 Measurement of CSF flow by phase contrast magnetic resonance imaging (PC-MRI)

Interest in patterns of CSF flow and what they reveal about the sites and rates of production and absorption of CSF have been rekindled by results obtained using PC-MRI and in particular measuring CSF flow through the cerebral aqueduct that connects the third and fourth ventricles. The development of PC-MRI has allowed real-time monitoring of blood flow in arteries and veins and, under favourable circumstances, also flow of CSF. MRI detects the magnetic dipoles of hydrogen atoms in small regions, called voxels, each of the order of 1 mm^3^. Application of this technique is more difficult for measuring CSF movements than for measuring blood flow because CSF movement is slower and the regions of interest are often small, containing only a few voxels. Despite these limitations, results of PC-MRI have provided useful information about CSF flow.

#### 4.2.1.2 Cyclic variations in CSF flow

From the results of PC-MRI and other techniques it is apparent that CSF flow in the cerebral aqueduct is not steady but rather varies over the cardiac cycle, driven by changes in blood volume in the brain vasculature [169]. In systole, more blood flows into the brain through arteries than leaves via veins while in diastole the reverse is true. Thus blood volume in the brain varies over the cardiac cycle and, because the skull is rigid and the inside volume is fixed, something else must compensate and this is achieved by shifting CSF from brain to spinal cord during systole and back during diastole. In normal people and experimental animals, a small part of this shift is movement of CSF in and out of the lateral and third ventricles via the cerebral aqueduct. Peak flow rates in each direction through the aqueduct of a normal subject are typically 10 times larger than the net average flow over the entire period. These fluid movements assist in mixing CSF in the ventricles and subarachnoid space.

#### 4.2.1.3 Direction of net flow

Early PC-MRI results for normal subjects were interpreted as yielding calculated net flows through the aqueduct consistent with values of CSF production rate in the ventricles determined by other means [169–173]. However, later studies have challenged the traditional view that CSF is produced primarily in the ventricles and absorbed primarily in locations distant from the ventricles.

#### 4.2.2 Changes in flow in hydrocephalus

Even before PC-MRI results became available there were reports showing that the standard story of a constant rate of CSF production in the ventricles and net flow from lateral and third ventricles outwards towards the subarachnoid space was not correct in patients with hydrocephalus, a condition where there is abnormal enlargement of the fluid-filled ventricles.

##### 4.2.2.1 Non-communicating hydrocephalus

In patients with non-communicating hydrocephalus the normal exit route for CSF from the ventricles, the cerebral aqueduct, is blocked. In these patients the rate of increase in ventricular volume has been reported to be far less than the normal rate of CSF production [174] and this can be reproduced in animal models. Some even contend that, at least in the cat, blockage of the aqueduct produces no ventricular swelling at all [175, 176]. Set against these reports must be experimental results (for an early example see e.g. [177]) and clinical experience (e.g. [135]) indicating that blockage does produce ventricular swelling. Milhorat *et al.*[178] tested this explicitly in monkeys by inflating a balloon in the fourth ventricle so blocking the aqueduct. Swelling of the lateral and third ventricles was seen to be initially rapid, possibly corresponding to the normal rate of production of CSF, but then to become less rapid. Three alternative explanations may be considered for why the rate of swelling following blockage of the aqueduct can be substantially less than that generally believed to be the “normal” CSF production rate: firstly secretion of CSF may have become inhibited; secondly blockage may not have been achieved; and lastly alternative routes for removal of CSF from ventricles may have been created or become important. Agreement has been reached [5, 9] that the CSF production rate does not change or at least does not change enough to explain the above result (e.g. [179] and for an early study see [174]). Change of CSF production rate will not be considered further here. Possible alternative routes for fluid escape will be considered in section 4.2.4.

##### 4.2.2.2 Communicating hydrocephalus and the possibility of reverse net flow

In communicating hydrocephalus, there is no impediment to CSF movement between ventricles and cisterna magna but there is still marked ventricular enlargement (by definition) and usually subarachnoid space contraction. PC-MRI measurements have shown greatly increased amplitudes of fluid flow through the aqueduct during the cardiac cycle, e.g 6-fold larger than normal, presumably because more CSF shifting from the cranium to the spinal cord must now come from the ventricles. At least one study comparing control subjects with patients with normal pressure hydrocephalus, found net flow in the expected direction and of the expected magnitude [180]. Some studies reporting large peak flows in each direction did not calculate a net flow [170, 181, 182] but there are now a number of reports of calculated net flows in the reverse direction, i.e. from fourth to third ventricle [69, 83, 84, 172, 183, 184].

**Figure 7:**
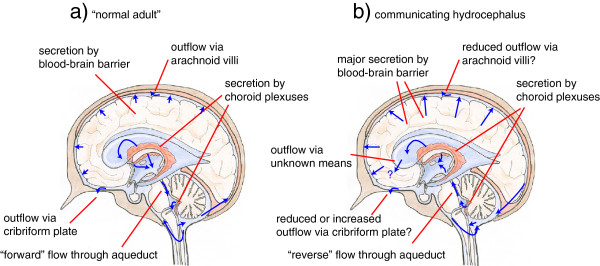
**View of net CSF flow pattern in normal adult brain compared with that proposed for communicating hydrocephalus. a)** In the normal adult, most of the CSF is secreted by the choroid-plexuses and is reabsorbed through the arachnoid villi or via the cribriform plate leading to the nasal mucosa. There is modest secretion across the blood-brain barrier into ISF most of which emerges into CSF across brain surfaces. The net flow through the cerebral aqueduct is from the third ventricle towards the fourth ventricle. **b)** In communicating hydrocephalus, the observation of reverse net flow through the aqueduct implies that formation of CSF occurs outside of the ventricles, probably by more extensive fluid secretion across the blood-brain barrier into the cortex and thence out into the CSF-containing spaces. Reverse net flow also requires some route for removal of CSF from the third and lateral ventricles. This route must accommodate both fluid secreted by the choroid plexuses located in these ventricles and fluid entering the third ventricle via the aqueduct. Possible routes are discussed in section 4.2.4. (The background image is the same as in Figure one-a taken from Strazielle *et al.* with permission [19]).

Suggestions of reverse net flow through the cerebral aqueduct in communicating hydrocephalus predate evidence from PC-MRI. Milhorat summarizing early results concluded (p139 in [135]) “In communicating hydrocephalus the normal subarachnoid pathways are obstructed and the subarachnoid flow is frequently reversed and directed into the ventricular system. … Diagnostically, retrograde filling of the ventricular system is a characteristic cisternographic finding in communicating hydrocephalus”. James and colleagues injected radiolabelled albumin into the cisterna magna of both dogs and monkeys with experimentally-induced communicating hydrocephalus and were able to see accumulation of the marker in the ventricles and periventricular regions, implying there must be net flow of fluid in the reversed direction, i.e. into the ventricles [185–188]. Such reverse net flow through the aqueduct implies both a major route for exit of CSF from the lateral and third ventricles other than the aqueduct and a major source of CSF other than the choroid plexuses (Figure 7). One proposal for an additional source is the cortical parenchyma, i.e. fluid secretion by the blood-brain barrier. Candidates for the route of outflow are less obvious. In conjunction with the ventricular enlargement there is periventricular oedema in white matter, which may allow abnormal routes for fluid removal from the ventricles [86].

#### 4.2.3 CSF flow in infants

Reverse flow in the aqueduct has also been reported by one group in children under two years; this being suggested as the normal condition for this age group [69, 84, 184]. There has also been a report of “early ventricular reflux” of radiopharmaceuticals in children under two years [189]. If net flow from fourth to third ventricle is accepted, “from where does it come, to where does it go” [69]? The blood-brain barrier can secrete fluid so the suggestion that the choroid plexuses are not the main source of CSF until two years is a hypothesis that should be tested. However, no evidence using any technique other than PC-MRI has been presented that would indicate a change in formation rate at or about the age of two. Using an infusion technique to determine the relation between intracranial pressure and rate of addition of fluid to CSF, Blomquist *et al.* found 588 ml/day as the control rate of CSF production, with no clear variation in age from two months to about ten years of age [190]. This is towards the high end of estimates of the normal adult rate. There is greater difficulty in understanding the fate of the CSF as discussed below.

#### 4.2.4 Possible alternative routes for CSF outflow from the ventricles

If net flow of CSF through the aqueduct is towards the lateral and third ventricles, then where does CSF leave these ventricles? Three possibilities need to be considered: a) there is another conduit for outward flow; b) CSF passes into parenchyma where it mixes with ISF and finally enters microvessels, i.e. it crosses the blood-brain barrier; and c) CSF flows into the parenchyma, mixes with ISF, and finally exits by other routes as discussed in section 4.1.

- Possible alternatives to the cerebral aqueduct as a conduit for flow of CSF out of the central ventricles have been discussed by Ghersi-Egea, Nagaraja, Fenstermacher and colleagues [130, 131]. These putative conduits are in regions where there is only a thin wall separating the ventricles from subarachnoid space [3]. However, there has been little support from any other groups for the idea that these regions do provide a pathway for flow. Nevertheless a search for such a conduit would be appropriate as there could be benefits from its presence as is seen in the treatment of hydrocephalus when an outflow route is produced surgically by third ventriculostomy (e.g. [114]). A further alternative route proposed by Milhorat [191] for flow out of the ventricles in hydrocephalic infants is via the choroid plexuses. This would require net flow to occur across the epithelium towards the blood, possibly via the paracellular route. However, Wislocki and Putnam found no movement of dyes via the choroid plexus in hydrocephalus induced by injection of lamp black into the cisterna magna [192] and Eisenberg, McLennan and Welch [193] found in kaolin-induced hydrocephalus that there was no reabsorption of fluid from the ventricles and thus perforce none via the choroid plexuses. However, Welch and Sadler had earlier found free diffusion of solutes as large as inulin from ventricles to blood but at a low rate as if across only a small fraction of the area of the choroid plexuses [194]. There was also an additional component of transport as if ventricular fluid were being moved across the choroid plexus by pinocytosis. The rate at which fluid was transferred by this route was later estimated to be at about 1/10th the normal rate of secretion [5]. In summary, while the balance of evidence is against a role for the choroid plexuses in net flow from ventricle to blood, the idea has not been conclusively excluded and requires further investigation.
- With regard to the second possible exit route for CSF, as already discussed in section 2.7, CSF absorption by penetration into the parenchyma and subsequent absorption into brain capillaries driven by colloid osmotic or hydrostatic pressures cannot be sustained for more than a few minutes so long as the blood-brain barrier is intact. It seems unlikely that this route is important for infants because there is no evidence of a major difference in the properties of their blood-brain barrier compared to older children. In hydrocephalus, alteration of the blood-brain barrier should be considered, but no evidence appears to have been produced that such alteration provides a route for the net removal of fluid from regions of the parenchyma. Linninger *et al.*[82] and Penn *et al.*[83] concluded that the blood-brain barrier was an important site of fluid removal from the parenchyma, but while their analysis supports the conclusion that there is some route of removal, the conclusion that this route is across the blood-brain barrier is the result of circular logic as their model for fluid movement considered only the blood-brain barrier as a possibility.
- Large scale flows of water in and out of parenchyma can be produced osmotically (section 2.7) and large fluid flows can also occur in regions of oedema. Thus in hydrocephalus, where there is periventricular oedema in white matter, it may well be the case that there are pathways for flow through the brain to alternative routes of exit such as those considered in section 4.1. Such a flow of fluid from ventricles into the parenchyma and thence to perivascular spaces was proposed as early as 1921 by Wislocki and Putnam [192]. By contrast, flow of the magnitude proposed through non-oedematous parenchyma from ventricles to the subarachnoid space would be without precedent. But “without precedent” only means that more direct evidence is required before the idea can be accepted.

Irrespective of the ultimate site of reabsorption of the fluid, if there is a marked increase in fluid transfer through the parenchyma there should be a marked change in the fate of markers injected into it. These are experiments that need to be done.

#### 4.2.5 Caveats on PC-MRI results for net flow through the aqueduct

The idea that in communicating hydrocephalus and in infants there is a cortical source of CSF and a sink for fluid in the ventricles via the parenchyma (or possibly the choroid plexuses) is based primarily on the validity of results for reverse net flow in the aqueduct. There are a number of features in the PC-MRI experiments, usually interpreted using proprietary software, which may not have been adequately tested and/or calibrated (for a recent review see [182]). Here are some of the pitfalls.

1. Flow measurements by PC-MRI can be calibrated in artificially controlled environments (so called “phantom” models) and they can detect time-dependent arterial blood flow with considerable accuracy as shown in both the brain and heart. However, it is not always clear that reported measurements have been calibrated under conditions appropriate to oscillatory CSF flow in the aqueduct (e.g. see [195] for discussion of some precautions).
2. To detect the slow flow in a small structure such as the cerebral aqueduct it is necessary to subtract the signals derived from a region that is thought to be stationary. Comparison of Bateman’s results with and without subtraction [84, 184] indicates that the subtraction is crucial. Is it sufficiently accurate? Note that it may not be possible to find a stationary region as the brain parenchyma moves in synchrony with the cardiac cycle [169, 196]. In any case, there is likely to be a net blood flow in an otherwise stationary region [197].
3. To calculate CSF flow from CSF velocity data obtained by PC-MRI, it is necessary to make assumptions about the flow profile across the aqueduct and/or to have enough voxels entirely within the aqueduct to calculate an adequate average. It has also been assumed that the cross-sectional profile of the aqueduct remains constant through the cardiac cycle. The available evidence suggests the calculations are sufficiently accurate for peak flows in both directions, but are they sufficiently accurate to calculate the smaller average over the entire cycle, i.e. the net flow?
4. The voxels to be included in the calculation of flow must be selected; a process which when automated is called segmentation. Has this process been sufficiently robust [173, 182]?

Calculating net flows from large flows in opposite directions suffers from the classic problem of subtracting large numbers to determine small differences; the signal can easily get lost in the noise. Experimentally there are large variations in the values of the net flows reported by various groups. Before the idea that reverse net flow occurs is finally accepted the reasons for these variations/discrepancies need to be better understood. At present the results for children are suggestive but not compelling. In communicating hydrocephalus the results from PC-MRI are supported by those from other techniques, notably cisternography and quantitative autoradiography [187, 198] which show that in these patients, but not in normal subjects, there is movement of radiolabelled albumin from cisterna magna to ventricles with accumulation in the ventricles and/or periventricular tissue. Furthermore in both communicating and non-communicating hydrocephalus there is oedema in the parenchyma adjacent to the ventricles, which may reduce resistance to fluid flow into the tissue from the ventricles. This might provide a route of fluid movement ultimately either to CSF in the subarachnoid space and thence to blood or lymph or more directly to sites outside the skull via perivascular or perineural spaces (section 4.1). It is also possible that the hydrocephalic state is associated with damage to or alterations in the properties of the blood-brain barrier or the choroid plexuses. There is a real need for experiments in which results from PC-MRI and from other techniques are compared directly in the same animals or patients.

### 4.3 Recent studies on perivascular routes for entry into and exit from the cortex

Proposals that substances enter and leave the parenchyma via perivascular spaces are as old as studies on the formation and absorption of brain extracellular fluids. Some aspects have been considered in earlier sections and earlier work has been reviewed elsewhere [9, 10]. It is clear that substances can move via vascular-associated routes. This section considers recent evidence obtained in rats and mice that has led to the well-publicised (e.g. [199] and [200]) glymphatic circulation proposal [201]: “ … CSF passes through the para-arterial space that surrounds arteries … and into the interstitial space … vectorial convective fluxes drive waste products away from the arteries and toward the veins. ISF and its constituents then enter the para-venous space. As ISF exits the brain through the paravenous route, it reaches lymphatic vessels in the neck, and eventually returns its contents to the systemic circulation”.

#### 4.3.1 The basis of the glymphatic circulation proposal: evidence from fluorescence imaging studies

Routes of transfer within mouse brain have been characterized by Iliff *et al.* using imaging of a number of different fluorescent markers [143]. For events near the cortical surface they used two-photon microscopy through a cranial window. For events occurring more than about 200 μm below the surface, movements were inferred from fluorescence imaging of brain sections. Iliff *et al*. found that when the 3 kDa labelled dextran, TR-d3, was included in closed-circuit ventriculo-cisternal perfusion of mice, it penetrated slowly into the parenchyma consistent with diffusion, just as expected from earlier results (section 2.8). By contrast when injected into the cisterna magna allowing access to the subarachnoid space, it distributed quickly to the cortical surfaces of the brain and within thirty minutes was seen prominently along the course of the arteries penetrating the cortex and also within the interstitium. Likewise 45 kDa ovalbumin-647, OA-647, also injected into the cisterna magna, followed the arteries to the region of observation and into the cortex but was not seen in the interstitium until later. Within the cortex, it was seen both in the periarterial space and within the smooth muscle layer of the arteries.

In a subsequent publication Iliff *et al.* emphasized that when the 3 kDA TR-d3 but also larger markers, 40 kDa dextran TR-d40 and 2000 kDa dextran FITC-d2000, were injected into the cisterna magna they each reached the region of observation along the walls of arteries [202]. Initially, however, they were not seen within the bulk of the CSF. The time-lapse images for TR-d40 (in the supplementary material of [143]) show particularly clearly that this marker followed the track of the arteries within the subarachnoid space but then spread out from these into the CSF. Such movement along arteries is consistent with the data obtained using MRI [203]. The route by which the markers gained access to the periarterial space from CSF in the subarachnoid cisternae is not known [202, 203]. The data show that the markers travelled rapidly along the arteries but also that, at least for markers of 45 kDa or less, they were able to move between the periarterial space and CSF, a manoeuvre which requires crossing the pial sheath (section 4.1). The idea that injected materials become associated with arterial walls has also been put forward by Rennels *et al.* for horseradish peroxidase [154, 156] and by Ghersi-Egea, Nagaraja, Fenstermacher and colleagues for sucrose [130, 131]. This retention presumably indicates a relatively low permeability of the pial sheath of the arteries to sucrose.

**Caveat:** 10 μl is a large volume to infuse into the CSF of a mouse and it is possible that the infusion may alter pressures and flows with the brain. Iliff *et al.*[202] note that in experiments under isoflurane anaesthesia, infusions like those used in their earlier work [143, 203, 204] “resulted in the non-physiological reflux of CSF tracer from the cisternal spaces into the fourth, third, and lateral ventricles” and they showed that even infusion at 1 μl min^-1^ for 10 min was associated with a temporary increase in intracranial pressure (ICP). They concluded that “Given that rapid paravascular CSF influx continues long after the normalization of these shifts in ICP, the paravascular CSF fluxes observed ....appear to represent physiological fluxes and not artefacts of changes in ICP resulting from CSF tracer infusion”. It is however conceivable that the increase in pressure might produce changes that outlive the increased pressure in the pial sheath of arteries traversing the CSF cisterns or even in the pial sheath of arteries within the cortex.

As found by Iliff *et al.* movement of fluorescent markers from periarterial spaces into the parenchyma depended upon their size [143]. Both 3 kDa TR-d3 and 45 kDa OA-647 spread into the parenchyma from both the cortical surface and the periarterial spaces surrounding the penetrating arteries, but 2000 kDa FITC-d2000 did not appear to spread at all even though it was clearly visualized in the periarterial space. These fluorescence data were not calibrated to allow quantitation of concentrations. However, from the comparison of the widespread and prominent distribution of 3 kDa TR-d3 seen using very sensitive fluorescence detection and the much less widespread distribution of 1 kDa gadolinium-DTPA detected using a less sensitive MRI-based method, Iliff *et al.* concluded that the concentrations of both markers in the parenchyma were much lower than those in the periarterial space near the cortical surface [203]. It is difficult to be quantitative about the transfer process from periarterial space to interstitium. In the case of TR-d3 the fluorescence signals from the periarterial regions may be saturated and reveal little about the concentrations while gadolinium-DTPA cannot be seen in the parenchyma.

The above fluorescence and MRI data are consistent with earlier findings concerning cortical penetration of horseradish peroxidase [79, 154–156] (cf. section 4.1) showing very rapid delivery of small quantities of markers into the parenchyma. From the speed of delivery over distances of millimeters it is apparent that transport along periarterial spaces cannot be just via diffusion (one point on which all are agreed [157]), i.e. both earlier and current data provide strong evidence for a convective component to the inward movement. These data are also consistent with studies on access of drugs to the parenchyma from CSF [158]. As reviewed by Papisov *et al.*, the evidence shows that drugs administered via the CSF are subject to “pulsation assisted translocation … into the perivascular space” [205]. The fluorescence results of Iliff *et al.* confirm and extend these findings and in addition, demonstrate that along the cortical surface movement of markers appears to be within a periarterial sheath, rather than just diffusely through the subarachnoid space [143].

Having reached the parenchyma, OA-647 (45 kDa) was seen in or on the walls of veins in the cortex 1 h but not 10 min after it was added to the cisterna magna. Iliff *et al.* interpret this as showing that the marker leaves the parenchyma via the veins [143]*.* A similar time sequence has been seen with another probe, rhod-2, consistent with this idea of progression [206]. The observed sequence, near arteries, near microvessels then near veins, is a major pillar of the glymphatic circulation proposal: fluid flow inwards along the arteries, then through the interstitium and thence outwards along the veins [143].

#### 4.3.2 Quantification of influx and efflux using radiotracers

Although fluorescence measurements are well suited for identifying pathways and are the most sensitive detection method that can be used in real time, they are difficult to make quantitative. Radiotracers have also been used by Iliff *et al*. to examine time courses for movement of markers [143]. They obtained data for uptake of mannitol (182 Da) and dextran-10 (10 kDa) injected into the cisterna magna and for retention of mannitol and amyloid β_1-40_ injected into the parenchyma. Their uptake curves for mannitol were interpreted as showing a single component with half-time of about 15-20 min. Their time course for retention of mannitol had a half-life of a few hours, perhaps similar to the half-life of about 4 h found for sucrose by Groothuis *et al.* as discussed in section 4.1. **Caveats:** by simple inspection of the sample uptake traces of Iliff *et al.,* it appears much more likely that the uptake of mannitol has two components, one very rapid, perhaps into the periarterial spaces, and another much slower, possibly into the parenchyma. Subsequent data reported for mannitol by Xie *et al*., (supplementary material in [207]), confirm that there are at least two components of the uptake. The earlier data comparing the uptakes of mannitol and dextran-10 are not extensive enough to allow any conclusion about the relative rates of uptake.

#### 4.3.3 Influences of aquaporin 4 (AQP4) located on astrocyte end-feet on perivascular flow

**Figure 8:**
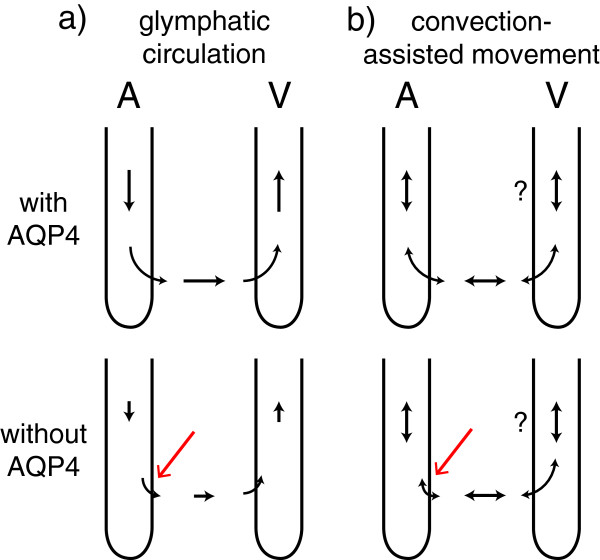
**Effect of absence of AQP4 on fluid flow along perivascular routes.** In both **a)** the glymphatic circulation proposal and **b)** the convection-assisted movement hypotheses described in Figure 9, the absence of AQP4 results in reduced movement of fluid from the periarterial spaces into the parenchyma (red arrow). In the glymphatic circulation proposal this would produce a build-up of fluid in the periarterial space, which would reduce flow into that space and would also remove the source of fluid destined for perivenous outflow. If instead, as in the convection-assisted movement hypotheses, fluid flow can occur in both directions in the periarterial spaces with little net flow, absence of AQP4 may not affect fluid flow along the periarterial space, a result more consistent with the data. A indicates the periarterial spaces, V the perivenous spaces. The question marks indicate that convection-assisted movement may or may not also occur in the perivenous spaces.

It is interesting to consider how perivascular fluid movement might be influenced by the presence of AQP4 located on glial end-feet enveloping blood vessels within the parenchyma [208]. Water and solutes moving between perivascular spaces and parenchyma must either be transported into the end-feet or go through the narrow spaces between them. AQP4 is abundantly expressed in the end-foot membrane facing towards the vasculature and thus is in a position to facilitate water transport via a transcellular route. It was found in AQP4 knock-out mice that entry of the smaller markers 3 kDa TR-d3 and 45 kDa OA-647 into the parenchyma from the periarterial space was greatly slowed [143]. This was interpreted by Iliff *et al.* as showing that by removing a pathway for water flow from periarterial space into interstitium the rate of glymphatic circulation was reduced. But reduction in glymphatic circulation should involve not only penetration into the parenchyma but also flow down the periarterial space (Figure 8) and yet data from Iliff *et al.* for the larger marker FITC-d2000 argues against such a reduction in periarterial convection: absence of AQP4 did not appear to affect penetration of the marker deep into the periarterial spaces. Thus the observation that AQP4 knock-out reduces movement into the parenchyma without reducing convection in the periarterial space presents the glymphatic circulation proposal with a substantial challenge. It indicates that convection in some manner assists movement along the periarterial spaces without producing a large net flow in one direction.

#### 4.3.4 Reassessment of the evidence: alternatives to the glymphatic proposal

**Figure 9:**
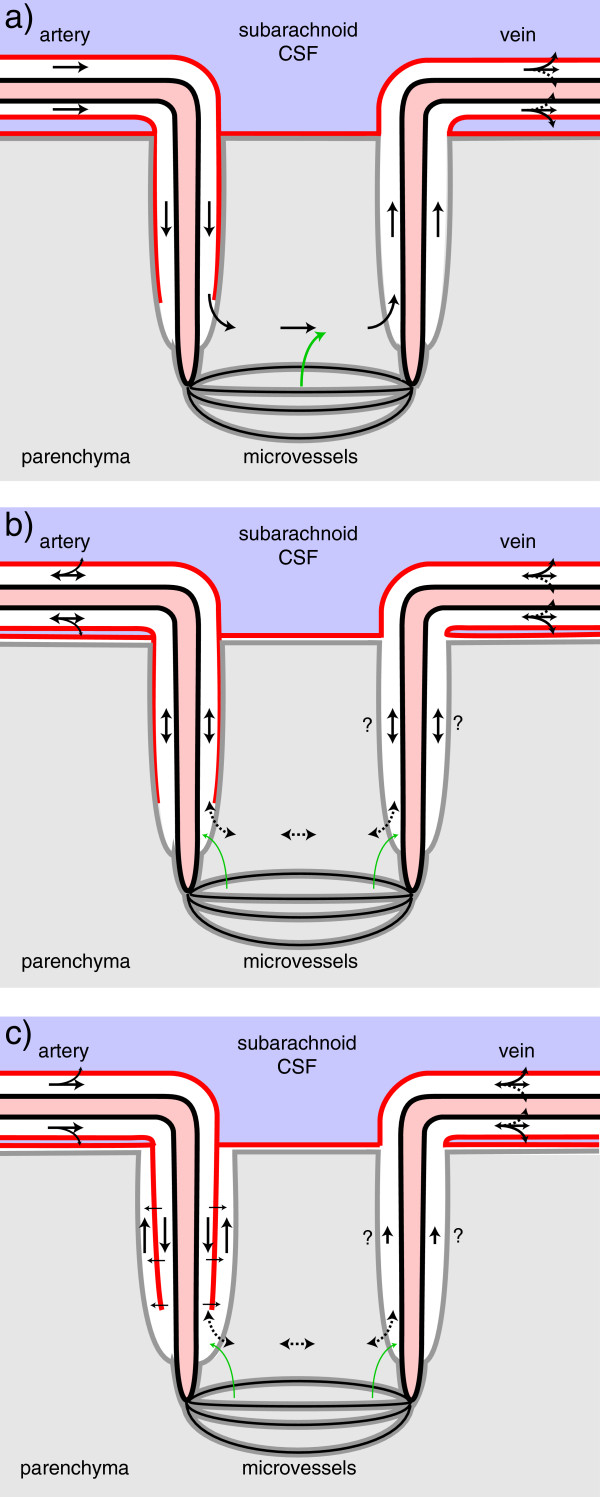
**Possible schemes to explain rapid influx of markers via periarterial spaces: a) the glymphatic proposal based on Figure five of Iliff** ***et al.*** **[**[143]**], b) stirring or mixing and c) layered flow.** In **a)** and **c)** there is preferential influx via the space between the arterial wall and the pial sheath (the inner periarterial space in Figure 6) while in **b)** convection back and forth, speeds up the rate of transfer of markers in both directions. Red lines represent pial membranes, grey lines the layer of glial end-feet or glia limitans, solid arrows are fluxes of markers carried or assisted by convection, dashed arrows are either by diffusion or assisted by convection and green arrows have been added as a reminder that fluid secreted by the blood-brain barrier contributes to the fluid in the parenchyma. The location of the pial barriers is based on Zhang *et al.*[23] (Figures 1d and 6). In **c)** the influx of fluid via the periarterial spaces may inflate the space of His providing a route for the return of fluid to the subpial space at the cortical surface and then to CSF in the subarachnoid space.

**Caveats:** Although the glymphatic proposal provides a basis for understanding much of the data, it does not explain the lack of effect of AQP4 knock-out on periarterial flux of the large dextran TR-d2000 discussed in the preceding section or the data for efflux via arterial routes discussed in section 4.1. So, are there alternatives to the glymphatic circulation proposal? In part, yes (Figure 9). Convection in the periarterial spaces within the cortex driven by cyclic changes in arterial pressure may produce either mixing (Figure 9b) or flows in opposite directions in different spaces of the arterial wall (Figures 6 and 9c). In either of these alternatives, convection assists movement into and out of the cortex without necessarily producing the net flow of fluid into the cortex as required by the glymphatic circulation proposal (Figure 9a). The back and forth periarterial convection resulting from the cardiac cycle is rapid on a time scale of seconds. Thus movement of a marker observed on a time scale of minutes would be averaged such that back and forth movements would not be detected only its net movement down its concentration gradient.

Can the alternatives explain Iliff *et al.’*s data [143]? The key observations are that when a marker was injected into the cisterna magna it was seen to enter the cortex along arteries and not veins whereas when the same marker was injected into the parenchyma, it was seen along veins but not arteries. These observations could be explained by the alternative hypotheses in terms of concentration differences and relative flow rates along arteries and veins. As shown in Iliff *et al.’*s data in the supplementary material [143] discussed earlier in this section, a marker injected into the cisterna magna would be delivered rapidly and at relatively high concentration to the periarterial spaces at the cortical surface but not the surrounding CSF or the perivenous spaces. Hence movement of a marker into the cortex is bound to be preferentially along arteries. By contrast when a marker is added to the parenchyma, a significant fraction of it might enter both perivenous and periarterial pathways but with mixing or outward movement being more effective in periarterial than in perivenous paths, the marker would be removed more rapidly from the periarterial spaces and the amounts seen there would be less. Thus the observations with the fluorescent markers, taken alone, can be rationalized. However neither the glymphatic circulation proposal nor the convection-assisted movement hypotheses can easily reconcile the observation that the fluorescent markers are preferentially located in perivenous spaces at late times with the data for preferential periarterial location and efflux of other markers discussed in section 4.1.

From both the more recent histological evidence ([23, 144], Figure 1c) and from the functional evidence discussed in sections 4.1 and 4.3, it can be concluded that the spaces or pathways involved in periarterial flow within the parenchyma are not in free communication with CSF in the subarachnoid space but are separated from it by at least the pial sheath or the pia mater. This separation is, however, challenged by the older observation that colloidal carbon gains access to a periarterial space from subarachnoid CSF [1]. There would appear to be three candidates for periarterial flow pathways within cortical parenchyma (Figure 6). One is the extracellular space in the smooth muscle layer as proposed by Weller and Carare (section 4.1). Another is the space between the smooth muscle layer and the pial sheath which Zhang *et al.* call the periarterial space [23] and is probably the Virchow-Robin space of the older literature (see [1] for discussion). The third is the space of His [1] that may exist immediately adjacent to the glial end-feet. Brinker *et al*. [17] label this outer space as the Virchow-Robin space. Because the space of His is continuous with the space below the pia mater at the cortical surface (Figure 1c) it could be called the perivascular subpial space.

It is tempting to speculate (see Figure 9c) that fluid arriving within the pial sheath of arteries in the subarachnoid space might be able to move inwards along the (inner) periarterial space between the smooth muscle and the sheath, much as proposed by Iliff, Nedergaard and colleagues. However, rather than this fluid continuing into the parenchyma (as in Figure 9a) it might inflate the space of His (an outer periarterial space) allowing fluid to return to the subpial space at the cortical surface and thence cross the pia mater to the subarachnoid CSF. The idea that the periarterial spaces may vary in calibre depending on local pressures is inherent in the idea that arterial pulsation drives convection. Such variability may be part of the explanation of the observation by Iliff *et al*. that there was far less inward movement of markers when the dura was not intact [143].

The alternative hypotheses, based on mixing or layers of flow as shown in Figure 9b and c, appear to explain the fluorescence data at least qualitatively. Furthermore, unlike the glymphatic circulation proposal, they can also accommodate the data considered in section 4.1 for periarterial efflux of markers (though not simultaneously preferential perivenous outflow of the fluorescent markers). The alternatives also provide an explanation for the lack of effect of AQP4 knockout on the movements of large markers into the periarterial space as they do not require net fluid movement from the periarterial space into the parenchyma.

The two alternative hypotheses indicated and the glymphatic circulation proposal are not mutually exclusive and furthermore there may be other scenarios. At present there is conflicting evidence for outward flow along veins and little evidence for *net* flow (as opposed to net marker flux down a concentration gradient) inwards along arteries. There is no evidence that the net flows, i.e. those inwards along the arteries, those proceeding through the parenchyma and those going outwards along the veins are of similar magnitude as required by the glymphatic circulation proposal. The implications of any of the schemes in Figure 9 which bring fluid deep into the cortex and then remove it, are that they would greatly shorten the diffusion distance from CSF to points within the parenchyma and hence the time taken for solutes to penetrate into or be cleared from regions of the cortex. The principle of operation in 9b and 9c is the same as that of a microdialysis probe which allows diffusional exchange between the interstitial fluid and the fluid within the probe.

### 4.4 Studies concerning the influence of sleep and anaesthesia on perivascular fluid flow and interstitial fluid volume

The glymphatic circulation proposal has been used to interpret experimental results on the effects of sleep and anaesthesia on perivascular fluid flow and interstitial volume in the parenchyma [143, 201].

#### 4.4.1 Changes in flow and volume inferred from rates of delivery of fluorescent markers

Xie *et al*. found that in awake mice the entry into the parenchyma of either of the 3 kDa fluorescent markers, FITC-dextran or TR-dextran, added via the cisterna magna was reduced compared to that seen in asleep or anaesthetized animals [207]. They interpreted their results as demonstrating the interstitial fluid volume to be less in the awake state. Some of their data, however, suggest another explanation. The data show that in the awake state the markers appear along the arteries in the subarachnoid space to a much smaller extent (or much later?) thus reduced penetration into the parenchyma would follow simply because the concentration in the periarterial space is lower. If this is the correct explanation, the observation that the amount reaching the interstitium was reduced reveals nothing about the interstitial spaces in the parenchyma. Although Xie *et al*. reported examples of data for concentrations measured simultaneously in the periarterial space and in a surrounding annulus of parenchyma, they did not report ratios of these concentrations [207]. If these ratios could be obtained they would provide information about the process of penetration of the markers into the interstitium and might allow conclusions about changes in interstitial volume.

#### 4.4.2 Interstitial fluid volume changes inferred from TMA iontophoresis measurements

Evidence that the interstitial fluid volume in the cortical parenchyma may be different in the awake state has been obtained [207] using the TMA iontophoresis method [46, 99] discussed in section 2.8. If there is no bulk flow and diffusion is isotropic (the same in all directions), the data from this method can be interpreted to yield an interstitial volume fraction and tortuosity. Xie *et al*. observed that the volume fraction estimated by the TMA method was higher if the rats were asleep than if they were awake. The transitions from the asleep to awake or awake to anaesthetized states were rapid, e.g. taking 15 min, (see supplementary material in [207]) and the inferred volume fraction changes were large, from 23 % to 14 % and 14 % to 23 % respectively. The experimental data are for regions within 300 μm of the cortical surface, but they are discussed by Xie *et al*. as if they apply to the entire cerebral cortex.

**Caveat:** As these changes are surprisingly large and fast it is necessary to ask if there are any confounding factors in the TMA measurements. Possible factors to consider include fluid flow, the structure of the extracellular space and voltage gradients with the associated patterns of current flow. For instance if there were fluid flow in the direction from the injection electrode to the measuring electrode in the awake but not in the sleeping mice this might account for a higher concentration of TMA reaching the recording electrode in the awake mice without any volume change. Alternatively a similar result might be seen if there were more flow in the opposite direction in the sleeping mice. However, to explain the data by such mechanisms would involve larger flows than are currently known to occur (Charles Nicholson and Tony Gardner-Medwin, personal communications) and would further require that these flows vary with behavioural state. (It should be noted, that the estimates of flows in the literature may be underestimates (sections 4.1 and 4.3.2) as they were based on measurements of outflow of markers from the cortex during barbiturate anaesthesia.) Changes of interstitial space configuration (for example opening or closing of diffusion blockages) seem also unlikely to account for the data, since they would be expected to affect the calculated tortuosity, which was not seen to vary significantly [207]. Thus it is hard at present to see a clear basis for challenging the inference of an altered space fraction for the regions within 300 μm of the cortical surface where the measurements were carried out (see Endnote^e^).

In a Physiological Society poster, Gardner-Medwin noted that in rats there is evidence against a large increase in ISF volume at the onset of sleep [209]. If there were to be an increase in volume the impedance of the tissue should decrease, but the only changes in impedance observed have been an increase associated with onset of rapid eye movement sleep and decrease on return to “normal” sleep [210, 211]. He also proposed for discussion several ideas based on movements of ions and water during slow wave sleep but at present additional experiments are needed to establish what effects these might have on TMA diffusion dynamics. Brinker *et al.* have focussed attention on possible factors other than altered glymphatic circulation that may have altered the width of interstitial spaces in animals exposed to the very artificial recording conditions, including changes in arterial blood pressure, venous blood pressure, stress hormone levels, and blood gases [17].

#### 4.4.3 Interstitial fluid volume changes inferred from radiotracer measurements

Xie *et al.* also used radiotracers to compare differences in interstitial fluid volume in awake, asleep or anaesthetized mice [207]. For this purpose they injected either radiolabelled inulin or amyloid-β into the cortex and followed the decrease in concentration over time. The half-time for the decrease was shorter for amyloid-β than for inulin probably reflecting the fact that amyloid-β is transported across the blood-brain barrier. The half-times for decrease for both substances were longer in awake mice compared to asleep or anaesthetized mice. Xie *et al.* interpreted these data (as with their fluorescence data, section 4.4.1) as implying smaller interstitial fluid volume and hence smaller rates of transfer within the interstitium in the awake mice. (For inulin the rate constants were roughly 0.008 min^-1^ awake compared to 0.016 min^-1^ asleep and 0.019 min^-1^ anaesthetized while for amyloid-β they were approximately 0.021 min^-1^, 0.05 min^-1^ and 0.045 min^-1^). **Caveats:** Although the changes in rate constants may reflect changes in interstitial fluid volume, many other things may have changed as well.

## 5 A current view of regulation of the extracellular fluids of the brain and their constant renewal

The composition of the extracellular fluids and the volume they occupy clearly have to be regulated to allow effective functioning of cells in the brain. This regulation is achieved by the presence of barriers that prevent free exchange between CNS and blood and mechanisms for production, distribution and reabsorption of fluid hence providing regular fluid turnover. The application of modern techniques to the study of the processes involved in brain fluid turnover has yielded new and important information. But in interpreting this information it is important not to neglect the basic anatomy and physiology of transport and flow. The underlying principle of control of total extracellular fluid volume in the brain is simple. Fluid is secreted into the brain at a rate that is relatively insensitive to changes in intracranial pressure (reviewed in [5, 9]). Fluid is returned from the brain to the blood at a rate which increases with intracranial pressure. The rate of fluid return increases with the extracellular fluid volume because increased volume increases intracranial pressure. At steady-state, when extracellular volume and intracranial pressure are constant, they take on whatever values balance inflow and outflow. This process has been considered extensively in earlier reviews [9, 16].

Regulation of the composition of extracellular fluid (section 1.4) depends primarily on the transport properties of the blood-brain barrier. This review has been concerned only with the evidence that the net process is a secretion and not with either the mechanisms or the importance of short-term local control. The net process must be secretion rather than filtration because the blood-brain barrier is an effective barrier. While hydrostatic pressures can drive water fluxes across the blood-brain barrier, unless these are accompanied by altered transport of solutes they will soon lead to concentration changes that produce osmotic pressures that oppose the net water fluxes (section 2.7). Viewed the other way round, solute fluxes, via specific transporters at the blood-brain barrier will produce osmotic changes that will force water to follow. These principles were established many years ago [7, 73] but some of their consequences have not been sufficiently widely appreciated. Importantly, there cannot be significant net reabsorption of fluid across the intact blood-brain barrier into the blood unless there is a mechanism for producing net movement of NaCl across the barrier in the direction from brain to blood and no such mechanism has been found or proposed.

Constant turnover of extracellular fluids has been widely viewed as necessary to provide a non-selective means to remove wastes for which there are no specific transporters. Continual secretion of “clean” fluid into the brain and reabsorption of whatever fluid is present by non-selective pressure-driven mechanisms achieves this end. Secretion of this “clean” fluid is partly the function of the choroid plexuses and partly the function of the blood-brain barrier. Perhaps surprisingly the relative contributions by each of these structures are still not known as their secretion rates are difficult to determine (sections 3.1.3 and 3.2). ISF flow which has been assumed to reflect blood-brain barrier secretion rate has been estimated from the rate of removal of markers from the brain but these measurements are called into question on two grounds: firstly they were performed with animals under barbiturate anaesthesia which may have inhibited both secretion and flow (sections 4.1 and 4.3.2) and secondly influx of CSF into the parenchyma via perivascular routes may contribute to ISF flow (section 4.3), which then does not reflect solely blood-brain barrier secretion. The reabsorption of fluid from brain eventually to blood occurs by a number of mechanisms (reviewed in [16]), all of which are relatively non-selective and increase in rate with intracranial pressure.

The broader outlines of brain fluid regulation were largely established in the older work. Much of more recent work has uncovered more complex fluid movements within the brain that have implications for the functions and regulation of the extracellular fluids. The results from PC-MRI measurement of CSF flow rates (section 4.2) leave no doubt that movement of CSF is much more extensive and variable than the slow steady flow envisaged in the classical account, i.e. secretion by the choroid plexuses and a sedate steady flow to exit sites. Results of some PC-MRI studies imply that *net* flow through the cerebral aqueduct is reversed so that the net flow is into rather than out of the third and lateral ventricles. Reversed flow has been proposed as the normal direction for net flow in children under two (in one study) and as the flow pattern in communicating hydrocephalus (in a number of studies). Reversal of flow has important implications, i.e. it requires that there is an important source of CSF other than the choroid plexuses and some means for removing fluid from the third and lateral ventricles other than via the cerebral aqueduct. In communicating hydrocephalus the route of removal may be created as a pathological consequence of the disorder but in children no plausible route has been proposed. Fluorescent tracer analysis has shown that flow can occur into the parenchyma along periarterial spaces. Whether or not this represents *net* fluid flow into the cortex and whether there is subsequent flow through the interstitium between arteriolar and venular ends of the microcirculation and *net* flow out of the cortex via perivenous routes, described as the glymphatic circulation, remain to be established (section 4.3).

## 6 Conclusion

Fluid is secreted into CSF by the choroid plexuses and into ISF at the blood-brain barrier. These secretions are driven by metabolic energy coupled to transport by the ubiquitous Na^+^, K^+^-ATPase, the Na^+^-pump. Fluid is returned to lymph and blood by a number of routes prominently including perineural pathways leading to the olfactory epithelium and the arachnoid villi. Fluid cannot be reabsorbed across the blood-brain barrier so long as the barrier remains intact. The relative rates of secretion by the choroid plexuses and at the blood-brain barrier remain uncertain. Similarly more data are required to be sure of the sources of fluid involved in turnover of ISF. Previous estimates of total CSF production rate including those made by the ventriculo-cisternal perfusion method may not include a large fraction of the contribution of ISF secretion at the blood-brain barrier. Earlier determinations of ISF production rates from measurements of the rate of efflux of markers from brain parenchyma may have underestimated the rate of fluid turnover by as much as 6-fold because experiments were performed under barbiturate anaesthesia, which may have suppressed both secretion and turnover. However, calculations of the rate of secretion at the blood-brain barrier made using measurements of rates of efflux of markers from the parenchyma may overestimate the secretion because fluid turnover mediating the marker efflux may include perivascular influx of CSF as well as blood-brain barrier secretion. If the observation of reversed net CSF flow through the aqueduct using phase contrast magnetic resonance imaging is substantiated, this would provide strong evidence that the blood-brain barrier is able to secrete fluid at a substantial rate compared to that of the choroid plexuses. Finally data showing net influx of CSF into the parenchyma via periarterial routes may indicate that local convection is occurring in periarterial spaces both within the subarachnoid spaces and within the cortex with or without associated flow through the parenchyma. The field is wide open for further experiments!

## Endnotes

^a^The Starling mechanism does not assume that proteins cannot cross peripheral capillary walls, only that they cross sufficiently slowly that they can exert almost their full osmotic pressure. The filtered fluid has concentrations of small solutes that are virtually the same as those in plasma, but the concentration of proteins is about 1/4 of that in plasma. At any point in time there is approximately the same amount of plasma protein in the tissues, equal to (average interstitial concentration of protein) × (total interstitial fluid volume), as in the plasma (plasma protein concentration in plasma) × (plasma volume). However, because the interstitial volume is about 4-fold bigger than the plasma volume there is still a marked difference in concentration and hence a marked colloid osmotic pressure across the membrane. In the central nervous system the microvascular permeability to protein is very much less than in the periphery and the plasma protein concentration in brain interstitium is very small.

^b^A unidirectional flux of water across a membrane, barrier or region is the flux of water molecules that start on one side and end up on the other. A unidirectional flux is normally measured using a tracer, e.g. for water often tritiated or deuterated water. Tracer molecules are added to one side of a membrane at a known fraction of the total number of water molecules on that side (including the tracer). The unidirectional flux of water is then calculated as the net flux of tracer divided by the fraction. The net flux of water is the difference between the unidirectional fluxes in the two directions.

For bulk flow as with the flow of blood along the length of a blood vessel, the unidirectional flux of water in the opposite direction to the flow is negligible and the unidirectional flux in the direction of flow is the same as the net flux. By contrast for pure diffusion down a gradient from a higher to a lower concentration, the ratio of the unidirectional flux in the same direction to the net flux is (higher concentration)/(difference in concentration) while the ratio of the unidirectional flux in the opposite direction to the net flux is (lower concentration)/(difference in concentration). For the actual process occurring across peripheral microvascular walls, the ratio of the back flux to the net flux is intermediate between the values for bulk flow and diffusion but much closer to that for diffusion. To put it another way, the movement of molecules against the flow in an artery can be ignored, but not the movements against the net flow across a capillary wall. The back flux of water is then almost as large as the forward flux.

^c^In most textbooks the story is told that in peripheral tissues there is filtration (a net movement out of the blood) of perhaps 1 % of the blood volume arriving in the tissue at the arterial ends of capillaries but that as a consequence of the drop in hydrostatic pressure along the capillaries there is reabsorption (a net inward movement) at the venous end of 90 % of that filtered leaving the 0.1 % of blood flow to become lymph. While this story uses the terms filtration and reabsorption correctly, it has been known for many years that it is rarely a correct description largely because it was based on outdated estimates of the interstitial forces and ignored changes in the hydrostatic and colloid osmotic pressures within the tissues and within the endothelial junctions. In those tissues with no other input of fluid, e.g. skin and muscle, filtration rather than absorption normally occurs over the entire length of the microvessels, capillaries and venules, with the overall amount filtered balanced by lymph flow. By contrast in tissues that have an input of a fluid with substantially less protein than plasma, e.g. the intestines during absorption of a meal or the cortex of the kidneys, there can be absorption of most of the added fluid into the vasculature while the rest carries interstitial protein into lymph. A similar process is thought to be occurring in the absorption of CSF that reaches the nasal mucosa (section 4.1).

^d^The inability of the intact blood-brain barrier to reabsorb interstitial fluid has been noted repeatedly. However from the frequency with which such reabsorption has been suggested, the point appears not to have been universally accepted. It may therefore be useful to provide a simple numerical calculation that emphasizes the difficulties. Imagine that CSF and ISF production containing 150 mM NaCl is 0.35 ml min^-1^ and that all of the reabsorption is occurring by pressure driven flow across microvessels in the parenchyma. If these have the normal properties of the blood-brain barrier, the reabsorbate will be nearly pure water, which by the hypothesis is being removed from the brain at 0.35 ml min^-1^. The net effect is that salt is being added by fluid production but is not being removed by fluid reabsorption. How long will it take to accumulate enough salt in the brain to stop the net reabsorption of water? The rate of addition of salt is 0.35 ml min^-1^ × 0.15 mmol ml^-1^ ~ 0.0475 mmol min^-1^. If the total water volume of the brain is taken to be 1 litre this amounts to a rate of change of concentration of 0.0475 mM min^-1^ which is a rate of increase of osmolality of 0.095 mOsmolal min^-1^. In other words each minute the salt build-up can offset a pressure of 0.095 mOsmolal * 19 mmHg mOsmolal^-1^ = 0.9 mmHg. In other words, *unless there is a means for removing the salt,* within a few minutes the osmolality of the brain will increase to offset any realistic hydrostatic (or colloid osmotic) pressure *difference* that could be driving the reabsorption. The difficulty with postulating pressure driven filtration of fluid across the blood-brain barrier either into the parenchyma or out of it is explaining how one moves the salt.

^e^The transitions from asleep to awake or awake to anaesthetized states reported by Xie *et al*. [207] are rapid, e.g. taking 15 min, and the inferred volume changes are large, from 23 % to 14 % and 14 % to 23 % respectively. They require addition to or removal from the interstitial spaces of both water and solutes as the fluid must remain nearly isosmotic with CSF and cells. It is difficult to imagine how the volume of the interstitium can be changed by so large a fraction so quickly unless the change is very localized.

To gain some appreciation of the size of the effects proposed consider the increase in ISF volume when the awake mice were anaesthetized. The same percentage change in ISF volume in humans if it occurred over the entire brain would require addition of about 90 ml of fluid containing about 300 mOsmoles of solutes per litre. These solutes cannot be K^+^ and Cl^−^ released from cells for the simple reason that, if maintained throughout a period of sleep, the increase in extracellular K^+^ concentration (to about 60 mM) together with the associated depolarization and sequelae would kill the cells. The solutes have to be primarily Na^+^ and Cl^−^. Indeed it would be very interesting to know if the NaCl content of brain extracellular fluid is different in the awake and asleep states as the volume is approximately proportional to this content.

The only possible mechanisms for NaCl to be added to the interstitial fluid are efflux from cells, fluxes across the blood-brain barrier or flow from CSF. The volume of the cells is much larger than the interstitial volume and thus even though the intracellular Na^+^ and Cl^−^ concentrations are small the cells may contain just about enough NaCl that if all were effluxed accompanied by water it might account for the shift. However, it is difficult to imagine how this could be made to occur. Secretion at the blood-brain barrier even if it were at the full rate needed for “normal” production of CSF (about 20 ml h^-1^) would be much too slow and there is no known mechanism for subsequent absorption of fluid by this route upon awakening. Furthermore introduction of fluid into the brain from the blood would be expected to increase intracranial pressure. That leaves exchanges with CSF. Can NaCl and water, about half of that in the entire CSF, be moved into the parenchyma from CSF in so short a time?

## Abbreviations

AQP4: Aquaporin 4
CSF: Cerebrospinal fluid
ISF: Interstitial fluid
MRI: Magnetic resonance imaging
PC-MRI: Phase contrast magnetic resonance imaging
TMA: Tetramethylammonium
